# Systematic review of the evidence sources applied to cost-effectiveness analyses for older women with primary breast cancer

**DOI:** 10.1186/s12962-022-00342-7

**Published:** 2022-03-01

**Authors:** Yubo Wang, Sean P. Gavan, Douglas Steinke, Kwok-Leung Cheung, Li-Chia Chen

**Affiliations:** 1 Centre for Pharmacoepidemiology and Drug Safety, Division of Pharmacy and Optometry, School of Health Sciences, Faculty of Biology, Medicine and Health, The University of Manchester, 1st Floor Stopford Building, Oxford Road, Manchester, M13 9PT UK; 2 Manchester Centre for Health Economics, Division of Population Health, Health Services Research and Primary Care, School of Health Sciences, Faculty of Biology, Medicine and Health, The University of Manchester, Oxford Road, Manchester, M13 9PL UK; 3 School of Medicine, University of Nottingham, Royal Derby Hospital Centre, Uttoxeter Road, Derby, DE22 3DT UK

**Keywords:** Economic evaluation, Decision-analytic modelling, Data sources of input parameters, Older women, Primary breast cancer

## Abstract

**Objective:**

To appraise the sources of evidence and methods to estimate input parameter values in decision-analytic model-based cost-effectiveness analyses of treatments for primary breast cancer (PBC) in older patients (≥ 70 years old).

**Methods:**

Two electronic databases (Ovid Medline, Ovid EMBASE) were searched (inception until 5 September-2021) to identify model-based full economic evaluations of treatments for older women with PBC as part of their base-case target population or age-subgroup analysis. Data sources and methods to estimate four types of input parameters including health-related quality of life (HRQoL); natural history; treatment effect; resource use were extracted and appraised. Quality assessment was completed by reference to the Consolidated Health Economic Evaluation Reporting Standards.

**Results:**

Seven model-based economic evaluations were included (older patients as part of their base-case (n = 3) or subgroup (n = 4) analysis). Data from younger patients (< 70 years) were used frequently to estimate input parameters. Different methods were adopted to adjust these estimates for an older population (HRQoL: disutility multipliers, additive utility decrements; Natural history: calibration of absolute values, one-way sensitivity analyses; Treatment effect: observational data analysis, age-specific behavioural parameters, plausible scenario analyses; Resource use: matched control observational data analysis, age-dependent follow-up costs).

**Conclusion:**

Improving estimated input parameters for older PBC patients will improve estimates of cost-effectiveness, decision uncertainty, and the value of further research. The methods reported in this review can inform future cost-effectiveness analyses to overcome data challenges for this population. A better understanding of the value of treatments for these patients will improve population health outcomes, clinical decision-making, and resource allocation decisions.

**Supplementary Information:**

The online version contains supplementary material available at 10.1186/s12962-022-00342-7.

## Introduction

Around half of the deaths from cancer occur in patients older than 70 years of age [1]. Breast cancer is the most prevalent cancer for females, and older patients may have different treatment goals than a younger population [2]. The increased likelihood of long-term comorbidities and frailty in this older population may preclude conventional treatment strategies (such as first-line surgery or adjuvant chemotherapy) due to the increased risk of treatment-related adverse events compared with younger people [3]. As a consequence, clinicians and decision-makers may be uncertain about the most appropriate way to manage these older patients [4]. Health economic evidence can inform treatment recommendations for breast cancer in older patients by comparing the incremental cost and health outcomes associated with different strategies available for this population [5]. However, robust evidence for the relative cost-effectiveness of the various treatment strategies observed in routine practice for older patients with breast cancer, including non-surgical intervention, is currently sparse.

Decision-analytic models are essential to produce this cost-effectiveness evidence by synthesising all relevant evidence and extrapolating expected cost and health outcomes over a lifetime time horizon [6]. As a minimum, health states such as 'disease-free', 'recurrence' (or 'progressed disease'), and 'dead' have been used previously to develop the structure of decision-analytic models for breast cancer [7]. This structural characterisation of disease is unlikely to vary by the age of diagnosis. However, there are few sources of evidence derived from older patients to populate the input parameter values of these decision-analytic models. The majority of randomised controlled trials (RCTs) of treatments for breast cancer, for example, have either excluded older patients due to their higher risk of morbidity and mortality or have recruited relatively low numbers of older patients [8]. Therefore, in the absence of data from older patients to populate key input parameter values, indirect evidence sourced from younger patients may be used instead to help estimate the cost-effectiveness of different treatment strategies for primary breast cancer in an older population.

Potential challenges may arise by using indirect evidence from younger patients if there are systematic differences with older patients in, for example, resource use, health-related quality of life (HRQoL), the natural history of the disease, or treatment benefits and harms. Older patients with breast cancer may have more interactions with the health care system and consume greater quantities of health care resources post-treatment than younger patients because of their relatively higher likelihood of comorbidity and frailty. Similarly, age-related comorbidities may result in older patients having relatively lower health-state utility values than younger patients [9]. The natural history of the disease may vary between younger and older patients if prognostic factors (such as endocrine receptor positivity) differ across age groups [10]. The magnitude and duration of benefit or direct harm from treatment (for example, the severity of adverse events after receiving chemotherapy) will likely depend on frailty experienced to a greater extent by older patients than younger patients [11].

In light of these potential differences between older and younger patients with primary breast cancer, if data from younger patients are used to populate input parameter values to estimate the expected cost and health outcomes of treatment strategies for older patients, analysts and decision-makers will need to appraise whether these sources of evidence are appropriate for the target population of the economic evaluation [12]. Inappropriate input parameter values may result in inaccurate cost-effectiveness estimates, decision uncertainty, and the value of undertaking further research for older patients. Therefore, this study aimed to appraise the sources of evidence and methods to estimate input parameter values in decision-analytic model-based cost-effectiveness analysis of treatments for primary breast cancer in older patients (≥ 70 years old). The results from this study were then used to inform recommendations to improve the estimates of key input parameters in future cost-effectiveness analyses of treatments for older patients with primary breast cancer.

## Methods

This study reports a systematic review of published economic evaluations of treatments (including surgery and any adjuvant or non-adjuvant treatments) for older females (≥ 70 years old) with early-stage primary breast cancer following the principles of the Preferred Reporting Items for Systematic Reviews and Meta-analyses Extension for Systematic Reviews (PRISMA) guidance [13]. This review focused on the methods used by the included economic evaluations to estimate four types of input parameters: (i) health-related quality of life (HRQoL), (ii) the natural history of the disease, (iii) the magnitude of relative treatment effects, and (iv) resource use.

### Eligibility criteria

The criteria for inclusion and exclusion in the systematic review were based on the PICO framework [14], i.e., Population (older women aged 70 years or more with early-stage primary breast cancer), Intervention (any treatment, including surgery with or without adjuvant therapy), Comparator (any therapy), Outcome (incremental cost and health outcomes), and Study design (full economic evaluation) (Table 1). A full economic evaluation is defined as "the comparative analysis of alternative courses of action in terms of both their costs and consequences" [15], including cost-effectiveness analyses (CEA), cost-utility analyses (CUA) and cost–benefit analyses (CBA) that used a decision-analytic model. Conference abstracts and manuscripts were written in a non-English language were excluded (Additional file 1: Appendix 1).

**Table 1:** Systematic review inclusion and exclusion criteria

Concepts	Inclusion criteria	Exclusion criteria
Population and conditions	Older women aged 70 years or more with (operable, Stage I, Stage II, or early) breast cancer	Only the aged below 70 years Only premenopausal women Only male breast cancer Only metastatic breast cancer Only locally advanced breast cancer Only recurrence of breast cancer Unconfirmed breast cancer Only non-invasive breast cancer Other diseases
Intervention	Surgery with/without adjuvant therapy	Head-to-head comparison Test to determine response after treatment Procedures for diagnosis of breast cancer Preventive strategy Preoperative therapy Nursing or rehabilitation care
Comparison	Any treatments	Treatments or prevention for adverse drug events Treating of cancer complication Follow up strategy
Outcome	Any outcome	Non-economic evaluation outcome, e.g., treatment preference or quality of life
Study Design	Full economic evaluation (CUA, CEA, CUA) that used a decision-analytic model in a peer-reviewed publication	Partial economic studies (cost of illness study, outcome description, cost description, outcome and cost descriptions, cost analysis) Systematic review Clinical trials, observational studies
Language	English	Other languages without English translation
Publication	Full-text article	Conference abstract or proceeding, abstract without full article Letter to editors, editorial, commentary, and news

### Information sources and search strategy

Ovid EMBASE® (1974 to 2021 Week 35) and Ovid Medline® (1964 to September 2021) were searched electronically from inception until September 2021. The search strategy (Additional file 1: Appendix 2) comprised disease-specific terms for early-stage primary breast cancer and terms to identify published economic evaluations according to the filters reported by the Centre for Reviews and Dissemination [16].

### Study selection and data collection

The titles and abstracts identified by the search strategy were screened independently for relevance against the inclusion criteria by two investigators (YW and LCC). The full texts of eligible studies were further retrieved and reviewed independently by two investigators (YW and LCC) to finalise study selection. At the full-text review stage, the age of the target population for the base-case analysis and, if relevant, for any age-specific subgroup analyses was identified within each economic evaluation to determine whether the study was designed for patients who were at least 70 years old. Discrepancies were resolved through consultation with a third reviewer (SG) to make a final decision.

### Data items

Data extraction comprised two stages. In the first stage, the following data were extracted from each economic evaluation by one author (YW): (1) study design (country; target population; strategies compared), (2) study characteristics (evaluation method, i.e., CEA or CUA; type of decision-analytic model; time horizon; perspective; health outcome measure used, and costs included), (3) evidence sources that were used to estimate four types of input parameter values (HRQoL; the natural history of the disease; relative treatment effect; and resource use/cost), (4) methods of analysis (whether deterministic/probabilistic sensitivity analyses or value of information (VOI) analyses were reported), and (5) estimated results (base-case and sensitivity analyses, VOI, and key drivers of relative cost-effectiveness through sensitivity analysis). In the second stage of data extraction, the characteristics of the estimation sample (sample size and mean age) were extracted from the original sources of evidence used by the included economic evaluations to estimate their input parameter values.

### Quality assessment

The completeness of reporting in each economic evaluation was assessed by 17 items in the Consolidated Health Economic Evaluation Reporting Standards (CHEERS) statement [17]. Full adherence to any item was noted as 'Yes', partially adherence was indicated as 'Partial', and non-adherence as 'No'. Two researchers (YW and LCC) independently appraised each identified economic evaluations' quality. Any discrepancies were discussed with a third reviewer (SG) to make a final decision. Quality assessment was summarised visually and reported by a narrative synthesis.

### Data synthesis

The extracted data from each economic evaluation were first reported in a table and summarised by a narrative synthesis. This summary described the sample of included economic evaluations according to the type of decision-analytic model used, the proportion of studies that had a target population of patients at least 70 years old in either the base-case or subgroup analysis, the treatment strategies compared, and the main results of each economic evaluation. For each economic evaluation, the sources of evidence used to estimate four types of input parameter were then appraised to determine whether they were obtained from an estimation sample that corresponded with the age of the target population (i.e., ≥ 70 years old). For the remainder of this study, (1) 'HRQoL' refers to the health state utility values, (2) the 'natural history of disease' refers to the probability of health events in the absence of a treatment effect, (3) the 'relative treatment effect' refers to the magnitude of difference between two treatments, and (4) 'resource use' refers to the direct health care resources consumed by patients. In the cases where evidence for input parameter values was based on an estimation sample of patients aged less than 70 years old, the methods of each economic evaluation were then appraised to determine whether any adjustment or calibration was performed to make these estimated values more appropriate for an older population.

## Results

**Fig. 1:**
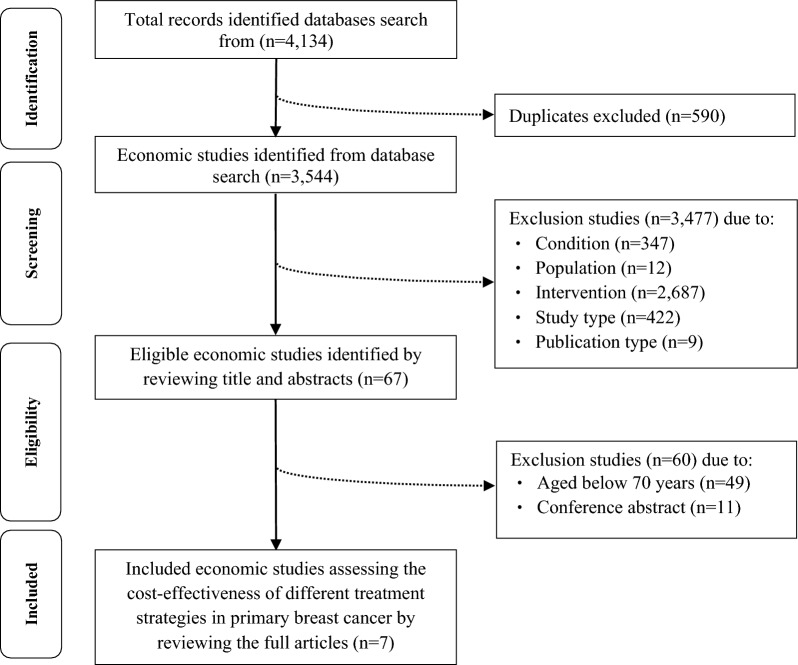
Selection of economic evaluations into this review

The PRISMA diagram (Fig. 1) illustrates the identification, screening and inclusion of studies. The electronic database searches identified 3544 studies, and 67 were read in full. The final sample comprised seven decision-analytic model-based economic evaluations of treatments for primary breast cancer in patients aged 70 years or more [18–24] (Fig. 1).

### Characteristics of included studies

All of the included economic evaluations reported both CEA and CUA. The decision-analytic models used by the identified economic evaluations included a cohort Markov model (n = 3) [20, 21, 24] and a patient-level simulation (n = 2) [22, 23]. Two studies did not report the type of decision-analytic model [18, 19]. All the seven economic evaluations used at least three health states within the structure of their decision-analytic model (disease-free; progressed disease; and dead). Different clinical outcomes were used between the economic evaluations to define the health state for progressed disease, including recurrence, local relapse, or metastasis. The structure of the decision-analytic model in four studies [21–24] also included an additional health state for treatment side effects (Table 2, Full data extraction in Additional file 1: Appendix 3).

**Table 2:** Summary of characteristics for included studies

Study, country	Target population	Type of model	Perspective, type of study	Intervention and Comparator	Results
Surgery plus adjuvant treatments used for comparisons					
Naeim et al. (2005) [18] USA	Subgroup analyses: 45,65, 75, 85 years women with early-stage node (+) breast cancer	Not stated	Health care provider CUA and CEA	Adjuvant chemo alone (CMF) Adjuvant chemo alone (AC) Adjuvant endocrine alone (Tamoxifen) Adjuvant Chemo (CMF) + Tamoxifen Adjuvant Chemo (AC) + Tamoxifen	Adjuvant endocrine treatment was cost-effective in older women
Naeim et al. (2005) [19] USA	Subgroup analyses: 45,65, 75, 85 years women with early-stage node (+) breast cancer	Not stated	Health care provider CUA and CEA	Adjuvant chemo alone (CMF) Adjuvant chemo alone (AC) Adjuvant endocrine alone (Tamoxifen) Adjuvant Chemo (CMF) + Tamoxifen Adjuvant Chemo (AC) + Tamoxifen	Adjuvant endocrine treatment was cost-effective in older women
Ward et al. (2019) [23] USA	Older women targeted: 70 years or older with estrogen-positive invasive breast cancer	Patient-level Markov microsimulation	Societal CUA and CEA	Adjuvant radiotherapy (APBI- alone) Adjuvant endocrine (Aromatase inhibitor alone)	Adjuvant endocrine treatment alone was the cost-effective strategy
Ward et al. (2020) [22] USA	Older women targeted: 70 years or older with estrogen-positive invasive breast cancer	Patient-level Markov microsimulation	Societal CUA and CEA	Adjuvant endocrine (Aromatase inhibitor alone) Adjuvant radiotherapy (APBI-alone) Their combination	Adjuvant endocrine treatment alone was the cost-effective strategy
Surgery as the comparator strategy					
Desch et al. (1993) [24] USA	Subgroup analyses: 60 to 80 years women with a diagnosis of primary breast cancer	Markov model	Societal CUA and CEA	Surgery alone Adjuvant chemotherapy alone	Adjuvant chemo was not a cost-effective treatment strategy for women aged more than 75 years
Skedgel et al (2013) [21] Canada	Subgroup analyses: 40, 50, 60, 70 and 80 + years women with T1bN0 breast cancer	Markov model	Direct payer CUA and CEA	Surgery alone Adjuvant chemotherapy alone Adjuvant chemotherapy + concurrent trastuzumab Adjuvant chemotherapy + sequential trastuzumab	Concurrent trastuzumab plus adjuvant chemotherapy was a cost-effective strategy
Sen et al. (2014) [20] USA	Older women targeted: 70, 75, and 80 years women with early-stage breast cancer	Markov model	Payer CUA and CEA	Surgery alone Adjuvant Radiotherapy EBRT Adjuvant Radiotherapy IMRT	EBRT was the cost-effective strategy

*CTx* chemotherapy, *RTx* radiotherapy, *ETx* endocrine therapy, *Trz* trastuzumab, *CUA* cost-utility analysis *CEA* cost-effectiveness analysis, *QALY* Quality-adjusted life year, *ICER* Incremental Cost-Effectiveness Ratio, *EBRT* External beam radiation therapy, *IMRT* Intensity-modulated RT, *APBI* accelerated partial-breast irradiation, *AC* adriamycin, cyclophosphamide, *CMF* cyclophosphamide, methotrexate, and 5-fluorouracil

Three economic evaluations (43%) had a base-case target population that focused exclusively on older patients aged ≥ 70 years [20, 22, 23]. The two studies by Ward et al*.* [22, 23] had a base-case target population of patients aged 70 years or older with estrogen-positive invasive breast cancer. Sen et al*.* [20] reported results for 70 years, 75-years, and 85-years old with early-stage breast cancer. Four economic evaluations (57%) reported cost-effectiveness estimates for older patients as part of subgroup analysis by age [18, 19, 21, 24]. The two studies by Naeim et al*.* reported results for subgroups of patients aged 75-years and 85-years old who had early-stage node-positive [18] and node-negative [19] breast cancer. Desch et al*.* [24] reported results for a subgroup of patients aged 60-years to 80-years old with a diagnosis of primary breast cancer, and Skedgel et al*.* [21] reported results for subgroups of patients aged 70 years and ≥ 80-years old.

Three studies [20, 21, 24] compared surgery alone with either adjuvant chemotherapy alone [24], radiotherapy [20], or chemotherapy ± trastuzumab [21]. The results from these three studies indicated that surgery alone was more cost-effective than surgery plus adjuvant treatments for the older population [20, 21, 24] (Table 2). Two studies compared surgery plus adjuvant chemotherapy with adjuvant chemotherapy ± endocrine therapy [18, 19]. Two studies compared surgery plus adjuvant radiotherapy with adjuvant endocrine therapy and their combination [22, 23]. Of these four studies, which compared different adjuvant strategies, the estimated results suggested that less adjuvant treatment, or less harmful adjuvant treatment (i.e., less intensive radiotherapy or less toxic chemotherapy), was more cost-effective for older patients with breast cancer [18, 19, 22, 23]. No published economic evaluation compared surgery with non-surgical treatment as the initial strategy to manage older patients with primary breast cancer (Table 2). In addition, no identified economic evaluation reported a value of information (VOI) analysis to investigate the need for further research to reduce uncertainty in the estimates of relative cost-effectiveness [25] (Table 2).

### Quality assessment

Table 3 reports the quality assessment of the seven economic evaluations according to the CHEERS criteria (Table 3). Thirteen domains of the CHEERS criteria (76%) were reported clearly by the included studies. However, in general, the economic evaluations whose base-case target population comprised older patients exclusively reported the sources of evidence to estimate input parameters more clearly than the economic evaluations that reported results for older patients as part of a subgroup analysis (Table 3). Six economic evaluations partially reported their analytical methods and study parameters which justifies the critical appraisal of these values for the remainder of this review.

**Table 3:**
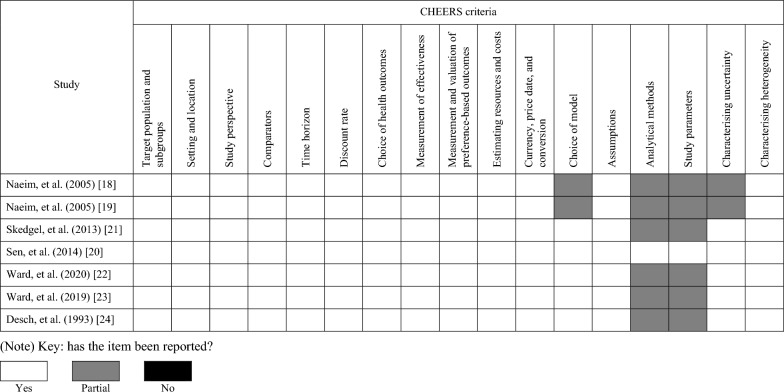
Reporting of each economic evaluation according to the Consolidated Health Economic Evaluation Reporting Standards (CHEERS) criteria

### Analysis of evidence sources for input parameters

The economic evaluations' sources of evidence and methods to estimate four types of input parameters (HRQoL, natural history, treatment effect, and resource use) are reported below. (Details of Input Parameters in Additional file 1: Appendix 4).

### Health-related quality of life

All seven economic evaluations reported expected health outcomes as quality-adjusted life years (QALYs) [18–24]. The EQ-5D instrument was used to estimate HRQoL in four studies [20–23]. Three studies estimated HRQoL values by expert elicitation [18, 19, 24]. Across the included economic evaluations, four approaches were taken to make the HRQoL values a function of the target population's age: (1) HRQoL, which was independent of age; (2) partial age-dependent HRQoL; (3) age-dependent HRQoL with a disutility multiplier; and (4) age-dependent HRQoL with an additive utility decrement (Table 4).

**Table 4:** Sources of evidence to estimate the health-related quality of life

Author, year	Health state	Instrument and data source	Target population	Sample size, mean age	Method of age adjustment
Without adjustment of age					
Naeim et al. (2005) [18] and Naeim et al. (2005) [19]	Disease-free Baseline Progression: hormone therapy minor toxicity with chemotherapy major toxicity with chemotherapy	Not reported Expert elicitation [67, 68]	45 years 65 years 75 years 85 years	150 Not reported	No
Desch et al. (1993) [24]	Disease-free Well Progression: First recurrence Side effect Minor toxicity with chemotherapy Major toxicity with chemotherapy	Not reported Assumptions	60 years 65 years 70 years 75 years 80 years	NA	NA
With adjustment of age					
Skedgel, et al. (2013) [21]	Disease-free: Disease-free baseline varied by age Progression: First local recurrence Second local recurrence Well after relapse Distant recurrence Side effect Congestive heart failure Febrile neutropenia AML/MDS Nausea/vomiting	EQ-5D-3L from previous literature [26] for baseline value Utilities for side effects: from the Cost-Effectiveness Analysis Registry without reporting data source	40 years 50 years 60 years 70 years 80 + years	2981, 74 years [26] Not reported for side effects	Partial adjustment: Age-dependent baseline values and fixed progression state values
Sen et al. (2014) [20]	1. Health states Disease-free: Surgery alone Surgery by different adjuvant treatments Progression: Recurrence Distant metastasis 2. Utility modifier 70–74 y 75–79 y 80–84 y > 85 y	1.EQ-5D from previous literature [27] 2.Standard gamble from previous literature [69]	70, 75, and 80 + years	97 patients with median age at 56 years [27]. Not reported [69]	Disutility multiplier to adjust standard gamble utilities by the mean age-dependent EQ-5D utilities in the general population
Ward et al. (2020) [22] and Ward et al. (2019) [23]	1. Utility Disease-free Baseline 2. Disutility value: Progression Distant metastasis Second malignancy: radiation Induced salvage mastectomy salvage axillary dissection after axillary recurrence Side effect Fracture Second malignancy: endometrial cancer salvage lumpectomy with radiation treatment of contralateral cancer Cardiac adverse event (MI) DVT Acute radiation dermatitis, Grade 3 Hot flashes Arthralgia Late radiation-induced fibrosis	1. Utility EQ-5D from a cross-sectional U.S. population survey 2005 [28] 2. Disutility from previous economic evaluation [70]	70 years or older	965 patients of a sub-cohort aged 65–74 years [28]	Age-dependent baseline values and health-state utilities with an additive utility decrement

*DVT* Deep vein thrombosis, *AML/MDS* acute myeloid leukaemia and/or myelodysplastic syndrome, *MI* myocardial infarction

The two studies by Naiem [18, 19] used HRQoL values fixed across age subgroups and independent of the target population's age. Patients were assumed to have lower utility if they received hormone therapy (HRQoL = 0.99), or chemotherapy with minor toxicity (HRQoL = 0.90) or major toxicity (HRQoL = 0.8). Similarly, Desch [24] also assumed that patients had the same utility values after experiencing minor and major side-effects from chemotherapy. This approach may overestimate the expected QALYs accrued by older patients if the loss of HRQoL due to treatment-related adverse events is greater than for younger patients.

Skedgel et al*.* [21] estimated HRQoL values, which were partially dependent on the age of the target population. The utility values for patients who were 'disease free' were calculated using EQ-5D data from the Medical Expenditure Panel Survey (MEPS) between 2000 and 2002 [26] (n = 38,678 adults). This approach enabled the authors to account for the negative association between age and HRQoL in the general population. However, the HRQoL values for subsequent health states (e.g., recurrence, second recurrence) and adverse events (e.g., nausea) appeared to be fixed and independent of age.

Sen et al*.* [20] used a disutility multiplier to estimate HRQoL values, which depended on the age of the target population. The MEPS (1998–99) was also used by Sen et al*.* to estimate age-dependent EQ-5D values for patients after successful treatment to preserve the negative association between age and HRQoL in the general population. Utility values for subsequent health states (e.g., local recurrence) were estimated from a published standard gamble study with 97 patients [27]. The authors then adjusted these utility values using a disutility multiplier based on the mean age-dependent EQ-5D values from the MEPS. This approach ensured that, on average, the HRQoL values accrued by patients who experienced these subsequent health states reflected the observed decline of HRQoL over their lifetimes. For example, the estimated HRQoL value for local recurrence was, therefore, lower for older patients than for younger patients.

Ward et al. [22, 23] used an additive utility decrement to estimate HRQoL values, which depended on the patient's age. A representative cross-sectional survey of the US population (n = 4,000) estimated a baseline EQ-5D value for 70 year old females between 2005 and 2006 [28]. The majority of subsequent health states had a corresponding disutility which was subtracted from this baseline EQ-5D value as an additive decrement (i.e., baseline HRQoL – disutility = new HRQoL). Similar to Sen et al*.*, this approach enabled the authors to estimate HRQoL values for patients who entered subsequent health states, which accounted for the lower HRQoL experienced by older patients, on average, compared with younger patients.

### Natural history of the disease

The included economic evaluations used four methods to estimate input parameters that reflected the natural history of breast cancer: (1) data were used from younger patients without adjustment; (2) data were used from older patients without adjustment; (3) plausible values were assumed and varied in a sensitivity analysis, and (4) data were used from younger patients and calibrated for an older population (Table 5).

**Table 5:** Sources of evidence to estimate the natural history of the disease

Author, year	Parameters used in studies	Data source	Age of target population	Mean age of estimation sample
From previous economic evaluations				
Skedgel et al. (2013) [21]	Disease-free to recurrence Proportion local recurrence/recurrence 'Instant' conversion from local to distant Side effects Rate of nausea |vomiting (grades 3 + 4) Rate of febrile neutropenia Rate of CHF Relative mortality risk |CHF Rate of AML/MDS Relative mortality rate |AML/MDS Relative risk of cardiotoxicity |conTZ Relative risk of cardiotoxicity |seqTZ	Recurrences from previous economic evaluations [71–73]. Adverse side-effects from previous trials [36, 37]	40 years 50 years 60 years 70 years 80 + years	Patients aged > 70 years account for 16% [36] Patients aged > 60 years account for 16.3% [37]
From randomised controlled trials				
Naeim et al. (2005) [19] and Naeim et al. (2005) [18]	Odds reduction of 10-year mortality Disease-free to death Adjuvant Chemo CMF Adjuvant Chemo AC Adjuvant Tamoxifen Adjuvant Chemo CMF + Tamoxifen Adjuvant Chemo AC + Tamoxifen	Background non-cancer mortality from United States life tables 1997 [74];	45 years 65 years 75 years 85 years	Age-specific mortality from 0 to 100 years
Sen et al. (2014) [20]	Disease-free to recurrence no RT Disease-free to recurrence + RT Recurrence to metastasis Metastasis to death	Clinical trial [29]	70, 75, and 80 years	> 70 years
Ward et al. (2020) [22] and Ward et al. (2019) [23]	Cumulative incidence Disease-free to death Overall survival Death from 2^nd^ cancer Disease-free to progression Ipsilateral breast tumours recurrence Contralateral breast cancer Distant metastasis Side effects Osteopenia requiring bisphosphonate Bone fracture Deep vein thrombosis Fibrosis/soft-tissue necrosis Hot flashes Arthralgia Radiation dermatitis, acute grade 3	Clinical trials [23, 29–35]	70 years or older	70 years [29] > 65 years [30] 65.7 years [31] 57 years [32] Not reported [33]
Desch et al. (1993) [24]	Disease-free to progression First recurrence	Clinical trials [38, 39]	60 years 65 years 70 years 75 years 80 years	48 years [38] Not reported [39]

*seqTZ* Sequential trastuzumab, *conTZ* concurrent trastuzumab, *AML/MDS* acute myeloid leukaemia and/or myelodysplastic syndrome, *CHF* chemotherapy-related congestive heart failure, *AI* Aromatase inhibitor, *APBI* Accelerated partial-breast irradiation, *AC* adriamycin, cyclophosphamide, *CMF* cyclophosphamide, methotrexate, and 5-fluorouracil

The two economic evaluations by Naiem et al*.* [18, 19] estimated the 10-year breast cancer-specific mortality for patients aged 75-years and 85-years old from studies where the estimation sample was younger (e.g., between 50 and 55% of the sample was below 55-years old). This approach may have underestimated the probability of death in the target population if the 10-year breast cancer-specific mortality is higher for older patients than younger patients. By contrast, Sen et al*.* [20] estimated the probability that patients experience health states (e.g., recurrence and metastasis) from published data of a trial that had recruited a sample of older females with breast cancer. It is likely that the probabilities derived from these trial data were more representative of an older population, given that the target population of Sen et al*.*'s economic evaluation and the estimation sample of the trial had similar patient characteristics.

Skedgel et al. argued that the prior probability of recurrence was unknown for their target population. The authors assumed a range of plausible values for the probability of recurrence across different ages to handle this. One advantage of this approach was that the impact of varying the probability of recurrence on cost-effectiveness estimates could be explored in a sensitivity analysis. Ward et al*.* [22, 23] estimated transition probabilities using data from a published trial that had whose sample was younger than 70 years old [23, 29–35]. To make these data more representative of an older population, the authors used calibration methods by applying a 'reduction factor' to the annual event rate in both arms of the trial. This approach reduced the absolute risk of events and made the input parameter values more appropriate for an older population.

### Magnitude of treatment effects

The economic evaluations used four methods to incorporate age-specific heterogeneity in the relative treatment effects. These methods include (1) direct estimation of age-specific treatment effects from RCT or meta-analysis data; (2) scenario analyses of plausible age-specific treatment effects in the absence of data; (3) the use of observational patient-level data to estimate age-specific treatment effects; and (4) the incorporation of age-specific behavioural parameters to modify the treatment effect.

Skedgel et al*.* [21] assumed that the relative treatment effect for adjuvant chemotherapy was a function of the patient's age. The authors estimated hazard ratios for 'premenopausal' (40 and 50 years) and 'postmenopausal' (60 and 70 years) patients using RCT data [36, 37]. These data indicated, for example, that adjuvant chemotherapy was less effective at reducing recurrence for older patients (HR: 0.672) than younger patients (HR: 0.563). The authors then assumed that the relative treatment effect for adjuvant trastuzumab was the same for both older and younger patients. Similarly, Desch et al*.* [24] assumed that the annual relative reduction in recurrence for patients aged 60 to 69-years old was 20%, compared with 30% for younger patients, according to data from a meta-analysis of RCTs [38, 39].

Naiem et al. [18, 19] first estimated the relative treatment effect of adjuvant therapies (odds-reduction of 10-year mortality) from a meta-analysis of RCTs for patients aged 45-years and 65-years old [40–43]. In the absence of evidence for the relative treatment effect in 75-years and 85-years old patients, the authors assumed three possible values (low, medium, and high) of treatment effects. In the 'high' scenario, the magnitude of the treatment effect was assumed to be equivalent to that for a 65-year old patient. The authors then estimated how reducing this treatment effect in older patients may impact cost-effectiveness estimates by using the 'medium' and 'low' scenario analyses. The details to calculate the medium values (extrapolated the trend of less benefit with increasing age) and the low values (minimal benefit) were not described explicitly.

Sen et al. [20] incorporated age-specific heterogeneity in the relative treatment effect by performing a patient-level analysis of data from the observational Surveillance, Epidemiology, and End Results (SEER) Program [44]. The authors estimated the 5-year and 10-year overall survival from radiotherapy compared with surgery. The estimated treatment effects were stratified by three age groups (70–74; 75–79; and 80–89 years old).

Ward et al. [22, 23] incorporated a behavioural parameter to reflect evidence that adherence to endocrine therapy may reduce in older patients. Data from a registry study of patients at least 65-years old estimated that compliance with endocrine therapy was 61% at 5-years. In the economic evaluation, this reduction of adherence had a subsequent impact on the relative effectiveness of endocrine therapy. By including this behavioural parameter, the authors were able to model potential changes in the relative effectiveness of treatment as patients became older.

### Resources and cost

The included economic evaluations used two methods to estimate input parameters for resource use: (1) estimated input parameters were independent of age, and (2) estimated input parameters were dependent on age (Table 6).

**Table 6:** Sources of evidence to estimate resource use

Author, year	Parameters used in studies	Data source	Age of target population	Mean age of estimation sample
Direct cost				
Naeim et al. (2005) [19] and Naeim et al. (2005) [18]	Treatments Adjuvant chemo alone (CMF) Adjuvant chemo alone (AC) Adjuvant endocrine alone (Tamoxifen) Adjuvant Chemo (CMF) + Tamoxifen Adjuvant Chemo (AC) + Tamoxifen	Published guidelines, research studies, and expert opinion of the treatment. Managing side effects of adjuvant chemotherapy from clinical trials [45]	45 years 65 years 75 years 85 years	Not reported
Skedgel et al. (2013) [21]	Treatment TC course FEC-D course 12 months adjuvant trastuzumab, per case Health states Local recurrence, per case Distant recurrence, per case Post-recurrence follow-up, per month Side effect Febrile neutropenia, per case AML/MDS, per month Chemo-related CHF, per month Chemo-related nausea and vomiting, per case Trastuzumab-related cardiotoxicity, per month Palliative trastuzumab, per case	TC course, FEC-D course, febrile neutropenia, AMD/MDS, and chemo related nausea and vomiting from previous literature [72, 73], 12 months adjuvant trastuzumab from previous literature [75], local recurrence, distant recurrence and post-recurrence follow-up from previous literature [47], chemo-related CHF from previous cost-effectiveness analysis [76], and palliative trastuzumab from literature [77]	40 years 50 years 60 years 70 years 80 + years	Not reported
Sen et al. (2014) [20]	Treatments No RT EBRT IMRT Brachytherapy Health states Recurrence, mastectomy Metastatic care Continued phase Death, last year of life	SEER-Medicare Previous costing study [78]	70, 75, and 80 years	70–74 years; 75–79 years; 80–94 years
Desch et al. (1993) [24]	Health states Chemotherapy, if given Side effects Minor toxicity Major toxicity	Previous literature [48, 49] Medical College of Virginia and estimates from Medicare data (1989)	60 years 65 years 70 years 75 years 80 years	Not reported
Direct and indirect cost				
Ward et al. (2020) [22] and Ward et al. (2019) [23]	Treatments Radiation Therapy Anastrozole (per year) Indirect costs of RT Indirect costs of Endocrine Therapy (Annual) Health states Salvage Mastectomy Salvage Lumpectomy or Axillary Dissection Metastatic Disease (per year)	ASCO and National Cancer Centers Network (NCCN) guidelines, all costs were adjusted to 2019 dollars using the US Bureau of Labor Statistics overall Consumer Price Index inflation	70 years or older	Not reported

*AC* adriamycin, cyclophosphamide, *CMF* cyclophosphamide, methotrexate, and 5-fluorouracil, *HRT* tamoxifen hormone therapy, *AWP* Average Wholesale Prices, *PHS* Public Health Service, *EBRT* external beam radiation therapy, *RT* radiation therapy, *IMRT* intensity-modulated RT

Five economic evaluations assumed that estimates of resource use were independent of each patient's age. Naeim and Keeler [18, 19] estimated the resource use for managing side effects of adjuvant chemotherapy (10% of patients needed treatment to manage low white cell counts, and 3% of patients required hospitalisation for neutropenic fever) based on data from an RCT that had a sample of younger patients (81% of the sample was ≤ 49-years old) [45]. However, this approach may have underestimated the resources required if hospitalisation rates or treatment for low white cell counts are higher in an older population [46]. Skedgel et al*.* [21] extracted the local and distant recurrence costs from published costing study [47]. These cost estimates were fixed for all age subgroups. The mean age of the sample in the published costing study was not reported, so it was not clear whether these data were applicable for a population of 70 year-old patients with primary breast cancer. Ward [22, 23] estimated direct and indirect costs using a hospital database and clinical guidelines. However, the authors did not report how the estimated cost of the metastatic disease ($23,460) was calculated.

Two economic evaluations estimated age-specific input parameter values for resource use [20, 24]. Desch [24] extracted cost data from the previously published economic evaluations [48, 49] and assumed that total costs of breast cancer treatment decreased as patients got older. This assumption was based on the reduction in follow-up costs, fewer late recurrences, and increased mortality from other causes over time. Sen estimated age-specific (70–74, 75–79, and 80–94 years old at diagnosis) cancer-related costs by conducting a matched cohort study from the SEER-Medicare database. Cancer patients were matched with non-cancer patients based on age, race, comorbidity, region, and year of diagnosis. All costs (inpatient, outpatient, physician, home health, hospice, and Durable Medical Equipment claims) for cancer and non-cancer patients were estimated over the 2-months before and up to 12-months after, date of diagnosis, and then stratified by type of initial treatment received. The cancer-related costs were the difference between the total cost accrued by cancer patients and their matched control [20].

## Discussion

Decision-analytic model-based economic evaluations will be essential to help inform the growing interest from decision-makers and clinicians about how best to treat older patients diagnosed with primary breast cancer. However, this review found just seven economic evaluations of treatments for this older population, and all studies compared adjuvant strategies only [18–24]. The authors of these economic evaluations used different methods to estimate input parameters values for HRQoL, the natural history of breast cancer, relative treatment effects, and resource use by using data from both older and younger patient populations. Therefore, a gap exists between the economic evidence required by decision-makers and the economic evidence available currently for managing primary breast cancer in older patients. To help close this evidence gap, the different methods to estimate age-specific input parameters reported in this review can inform the design of future model-based economic evaluations and strategies to overcome the relative scarcity of data from older patients.

A key distinction between the identified economic evaluations was whether they reported cost-effectiveness evidence for older patients as the base-case analysis or as part of subgroup analysis. For example, over half (57%) of economic evaluations in this review reported a subgroup analysis for patients older than 70 years old. Subgroup analyses in economic evaluations are a valuable method to investigate heterogeneity in cost-effectiveness by identifiable patient characteristics [50]. However, it is vital to ensure that input parameter values are appropriate for each subgroup under investigation. If future economic evaluations report age-specific subgroup estimates for older patients with primary breast cancer, decision-makers and analysts should appraise whether the input parameter values are expected to vary across age groups or whether they are independent of age. This will improve the face validity of the model-based analysis and the external validity of the subgroup estimates of cost-effectiveness.

The National Institute for Health and Care Excellence (NICE) Decision Support Unit and the International Society for Pharmaceutical Outcomes Research advise that HRQoL should be estimated using evidence from a population that is similar (e.g., age, sex, and disease severity) to the modelled population [51, 52]. Age is a key determinant of HRQoL because older patients may have lower values than younger patients due to comorbidities and frailty [53]. In this review, the methods used by Ward et al. [22, 23] and Sen et al. [20] to estimate HRQoL (i.e. disutility multipliers or additive utility decrements informed by baseline values from representative surveys of the general population) are helpful techniques for future economic evaluations to incorporate age-specific input parameter values when only data from younger patients are available. Peasgood et al*.* [54] report a systematic review and meta-regression of health state utility values for breast cancer. Many economic evaluations have previously obtained relevant input parameter values from this study. This meta-regression could be developed further by investigating whether including the mean age of the patients in each study affects the estimated relationship between health state utility and the other variables. Similarly, some studies have used mapping methods to estimate the statistical association between breast cancer-specific patient-reported outcomes (e.g., the European Organization for Research and Treatment of Cancer Quality of Life Questionnaire, EORTC QLQ-C30 [55]) and generic instruments used to estimate health state utility values (e.g., EQ-5D) [56–58]. Future mapping studies could build on these earlier studies by including an age variable to account for the impact of older age on HRQoL.

Depending on the decision problem, older patients' natural history of breast cancer may be available to estimate transition probabilities between health states. However, most economic evaluations in this review used evidence from a younger population to estimate these natural history parameters. The calibration method by Ward et al. [22, 23], which adjusted estimates from younger patients so that they were appropriate for an older population, is a helpful technique for future economic evaluations when data from patients over 70 years old are not available. Alternatively, future economic evaluations could use formal expert elicitation methods [59] to estimate these natural history parameters with sensitivity analyses around plausible values. There has also been an increase in the availability of linked primary care, secondary care, mortality, and cancer register data sources [60]. These linked data could also be a valuable source of evidence to estimate the natural history of breast cancer for older patients in routine practice, ensuring that any selection bias and confounding are accounted for.

The magnitude of the estimated relative treatment effects and toxicity for patients with breast cancer can vary by age and type of treatment [61]. For example, chemotherapy and radiotherapy have shown a limited survival benefit for older patients with primary breast cancer than their younger counterparts [61]. Similarly, the effectiveness of endocrine and biological therapy is highly associated with the level and sensitivity of hormone receptors and HER-2 receptors. Older patients have a higher level of ER and PR receptors, whereas a lower level of HER-2 receptors [62, 63]. For future economic evaluations, the target population should be defined clearly in terms of whether a patients' age interacts with the biological mechanisms of disease and, as a consequence, whether the estimated treatment effects are appropriate for that population. The choice of comparator strategies should also be limited to relevant ones for the target population in routine practice. For example, non-surgical strategies may be the most appropriate comparators for patients who are ineligible for surgery due to frailty.

Older patients consume more health care resources than younger patients [63, 64] and costing studies from the US and UK [65, 66] indicated that the main cost drivers for cancer treatment in older populations were from treating side effects and related health care (for example, care and management of chemotherapy-induced neutropenia, radiotherapy-induced skin/gastrointestinal reaction, and trastuzumab induced cardiotoxicity). It is essential for future economic evaluations of treatments for primary breast cancer to report the evidence sources for resource use transparently to help decision-makers appraise whether these data can generalise to an older population. Sen [20] undertook a matched cohort study to estimate the incremental cost of managing older patients with primary breast cancer. Future research could use a matched cohort design to estimate valuable resource use data for older patients with primary breast cancer using large national observational datasets which link secondary care resource use with cancer diagnosis data. These patient-level data could then provide a better characterisation of how parameter uncertainty in estimates of resource use is distributed.

One limitation of this review was that the search strategy only identified published economic evaluations from peer-reviewed academic journals and may have missed some economic evaluations in the grey literature from government or private organisations. However, the sample of included studies successfully identified a broad range of different methods used to estimate input parameter values for an older population. A second potential limitation was that this systematic review focused only on four specific input parameter types. Therefore, valuable methods to estimate other input parameter types may have been omitted. However, the focus on input parameters for HRQoL, the natural history of the disease, treatment effects, and resource use was sufficient to characterise the majority of essential input parameters for any model-based cost-effectiveness analysis.

Future research could begin to estimate the cost-effectiveness of the different strategies along the full pathway of care observed in routine practice to manage older patients with breast cancer. This economic evidence would be valuable to inform how best to treat these patients by simultaneously considering health outcomes and costs to the healthcare system. Future research could also undertake a value of information analysis, based on the probabilistic outputs from these model-based analyses, to establish whether subsequent primary research in older patients would be worthwhile to reduce uncertainty in estimates of cost-effectiveness. Finally, future research could appraise the sources of evidence and methods to estimate input parameter values within economic evaluations of treatments for older patients diagnosed with other types of primary cancer.

## Conclusion

The number of patients older than 70 years of age diagnosed with primary breast cancer is increasing. Health economic evidence will be essential to inform how best to manage these patients. This systematic review found only seven CEAs for this older population, indicating that further economic evidence will be valuable to meet the needs of decision-makers and service commissioners in the future. The methods to estimate input parameters described in this systematic review can help analysts overcome common data challenges to improve the accuracy of expected cost and health outcome estimates. Well-designed observational studies using national register data and formal expert elicitation exercises also present a considerable opportunity to improve the quality of input parameters estimates for this older patient population. A greater emphasis on understanding the cost-effectiveness of care for older patients with primary breast cancer will simultaneously improve population health outcomes, clinical decision-making for these patients, and the allocation of limited resources for health care.

### Supplementary Information

**Additional file 1: Appendix 1**. Reasons for excluding studies. **Appendix 2**. Search Strategy for Databases. **Appendix 3**.Full Data Extraction Tables. **Appendix 4**. Details of Input Parameters.

## Data Availability

Supplement information is included in the appendices.
