# Supplementary material for: Systematic review of the evidence sources applied to cost-effectiveness analyses for older women with primary breast cancer
Source: Cost Eff Resour Alloc. 2022 Mar 1;20:9. doi: 10.1186/s12962-022-00342-7 (PMC8889747; doi:10.1186/s12962-022-00342-7)
Supplement: Supplementary file 1 — Additional file 1: Appendix 1. Reasons for excluding studies. Appendix 2. Search Strategy for Databases. Appendix 3.Full Data Extraction Tables. Appendix 4. Details of Input Parameters. [file 12962_2022_342_MOESM1_ESM.docx]

**Sources of Evidence in Cost-Effectiveness Analyses for Older Women with Primary Breast Cancer: A Scoping Review and Future Directions**

**Supplementary Appendix**

[Supplementary Appendix 1. Reasons for excluding studies 2](#_Toc93407216)

[Supplementary Appendix 2. Search Strategy for Databases 3](#_Toc93407217)

[Supplementary Appendix 3. Full Data Extraction Tables 6](#_Toc93407218)

[Supplementary Appendix 4. Details of Input Parameters 13](#_Toc93407219)

[Reference 26](#_Toc93407220)

# Supplementary Appendix 1. Reasons for excluding studies

|  | **Exclusion Criteria** | **Number of excluded studies** |
| --- | --- | --- |
| **Condition** | Metastatic breast cancer | 163 |
|  | Non-invasive breast cancer | 115 |
|  | Other diseases | 69 |
| **Population** | Younger patients aged < 70 years | 49 |
|  | Premenopausal women only | 12 |
| **Intervention** | Biopsy strategy or outcomes | 175 |
|  | Adherence | 118 |
|  | Diagnostic test to determine the response | 327 |
|  | Follow-up | 182 |
|  | Genetic test | 135 |
|  | Healthcare | 123 |
|  | Neoadjuvant treatment or preoperative care | 118 |
|  | Nursing or exercise or rehabilitation care or psychological care | 117 |
|  | Disease prevention and prediction | 217 |
|  | Prognosis observation | 380 |
|  | Screening and imaging strategy | 217 |
|  | Lymph node exam or excision | 175 |
|  | Side effect control | 89 |
|  | Treatment outcome assessment | 156 |
|  | Patient satisfaction | 104 |
|  | Sociodemographic comparison | 54 |
| **Type of study** | Statistical analysis or case control study | 177 |
|  | Guideline and audit | 20 |
|  | Partial economic evaluation | 211 |
|  | Clinical trial-based study | 14 |
| **Type of publication** | Non-full paper | 5 |
|  | Review | 4 |
|  | Conference abstract | 11 |

# Supplementary Appendix 2. Search Strategy for Databases

1. **Search strategy for Ovid MEDLINE(R) 1946 to September Week 2 2021**

| 1 | Economics/ |
| --- | --- |
| 2 | exp "Costs and Cost Analysis"/ |
| 3 | Economics, Dental/ |
| 4 | exp Economics, Hospital/ |
| 5 | Economics, Pharmaceutical/ or Economics, Medical/ or Economics, Nursing/ |
| 6 | value for money.ti,ab. |
| 7 | budget$.ti,ab. |
| 8 | (economic$ or cost or costs or costly or costing or price or prices or pricing or pharmacoeconomic$).ti,ab. |
| 9 | (expenditure$ not energy).ti,ab. |
| 10 | 1 or 2 or 3 or 4 or 5 or 6 or 7 or 8 or 9 |
| 11 | ((energy or oxygen) adj cost).ti,ab. |
| 12 | (metabolic adj cost).ti,ab. |
| 13 | ((energy or oxygen) adj expenditure).ti,ab. |
| 14 | 11 or 12 or 13 |
| 15 | 10 not 14 |
| 16 | letter.pt. |
| 17 | editorial.pt. |
| 18 | historical article.pt. |
| 19 | 16 or 17 or 18 |
| 20 | 15 not 19 |
| 21 | Animals/ |
| 22 | Humans/ |
| 23 | 21 not (21 and 22) |
| 24 | 20 not 23 |
| 25 | exp Breast Neoplasms/ |
| 26 | exp Breast/ or exp Breast Diseases/ |
| 27 | exp Neoplasms/ |
| 28 | (cancer$ adj3 breast$).tw. |
| 29 | (neoplas$ adj3 breast$).tw. |
| 30 | (carcinoma$ adj3 breast$).tw. |
| 31 | (adenocarcinoma$ adj3 breast$).tw. |
| 32 | (tumour$ adj3 breast$).tw. |
| 33 | (tumor$ adj3 breast$).tw. |
| 34 | (malignan$ adj3 breast$).tw. |
| 35 | 26 and 27 |
| 36 | 28 or 29 or 30 or 31 or 32 or 33 or 34 |
| 37 | 25 or 35 or 36 |
| 38 | limit 37 to female |
| 39 | Premenopause/ |
| 40 | 38 not 39 |
| 41 | exp Mastectomy/ |
| 42 | exp Surgical Oncology/ |
| 43 | (mastectom$ or operat$ or surg$ or (breast adj conserv$) or lumpectom$ or "wide local excision" or segmentectom$ or mammectom$ or quadrantectom$). ti,ab. |
| 44 | 41 or 42 or 43 |
| 45 | 24 and 40 and 44 |

1. **Search strategy for Ovid Embase 1974 to 2021 Week 35**

| 1 | health-economics/ |
| --- | --- |
| 2 | exp economic-evaluation/ |
| 3 | exp health-care-cost/ |
| 4 | exp pharmacoeconomics/ |
| 5 | 1 or 2 or 3 or 4 |
| 6 | (econom$ or cost or costs or costly or costing or price or prices or pricing or pharmacoeconomic$).ti,ab. |
| 7 | (expenditure$ not energy).ti,ab. |
| 8 | (value adj2 money).ti,ab. |
| 9 | budget$.ti,ab. |
| 10 | 6 or 7 or 8 or 9 |
| 11 | 5 or 10 |
| 12 | (metabolic adj cost).ti,ab. |
| 13 | ((energy or oxygen) adj cost).ti,ab. |
| 14 | ((energy or oxygen) adj expenditure).ti,ab. |
| 15 | 12 or 13 or 14 |
| 16 | 11 not 15 |
| 17 | animal/ |
| 18 | exp animal experiment/ |
| 19 | nonhuman/ |
| 20 | (rat or rats or mouse or mice or hamster or hamsters or animal or animals or dog or dogs or cat or cats or bovine or sheep).ti,ab,sh. |
| 21 | 17 or 18 or 19 or 20 |
| 22 | 16 not 21 |
| 23 | breast tumor/ |
| 24 | breast/ or breast disease/ |
| 25 | neoplasm/ |
| 26 | 24 and 25 |
| 27 | (cancer$ adj3 breast$).tw. |
| 28 | (neoplas$ adj3 breast$).tw. |
| 29 | (carcinoma$ adj3 breast$).tw. |
| 30 | (adenocarcinoma$ adj3 breast$).tw. |
| 31 | (tumour$ adj3 breast$).tw. |
| 32 | (tumor$ adj3 breast$).tw. |
| 33 | (malignan$ adj3 breast$).tw. |
| 34 | 27 or 28 or 29 or 30 or 31 or 32 or 33 |
| 35 | 23 or 26 or 34 |
| 36 | limit 35 to female |
| 37 | premenopause/ |
| 38 | 36 not 37 |
| 39 | radical mastectomy/ or partial mastectomy/ or simple mastectomy/ or modified radical mastectomy/ or subcutaneous mastectomy/ or mastectomy/ or "patient history of mastectomy"/ or extended radical mastectomy/ or prophylactic mastectomy/ |
| 40 | cancer surgery/ or surgical oncology/ |
| 41 | (mastectom$ or operat$ or surg$ or (breast adj conserv$) or lumpectom$ or "wide local excision" or segmentectom$ or mammectom$ or quadrantectom$).ti,ab. |
| 42 | 39 or 40 or 41 |
| 43 | 22 and 38 and 42 |

# Supplementary Appendix 3. Full Data Extraction Tables

Table S1. Full Data Extraction Table for [Naeim A. and Keeler E. B. (2005) [1]](#_ENREF_1)

| **Study Design** | **Study Characteristics** | **Data Sources** | **Analysis** | **Results** |
| --- | --- | --- | --- | --- |
| **Target population:**  65-year-old women to 75-and 85-year-old women with early-stage node (-) breast cancer  **Alternatives:**  Surgery with five treatment options were considered:  (a) Cyclophosphamide, methotrexate, and 5-flurouracil (CMF) × 6 chemotherapy,  (b) Adriamycin, cyclophosphamide (AC) × 4 chemotherapy,  (c) Tamoxifen hormone therapy (HRT)-5 years,  (d) Tamoxifen (HRT)-CMF,  (e) Tamoxifen (HRT)-AC.  **Country:**  USA | **Evaluation method:**  CEA  **Model type:**  Not stated  **Time horizon:**  10 years  **Perspective:**  health care provider  **Benefit measure:**  QALY  **Direct costs included:**  initial treatment  **Indirect costs included:**  Not applicable | **Effectiveness:**  benefits of adjuvant therapy: meta-analyses conducted by the EBCTCG [[2-5](#_ENREF_2)] Transition probability: 1999 mortality data from the National Centre of Health Statistics. Economic data  **Health-related quality of life:**  previous reviews[[6](#_ENREF_6), [7](#_ENREF_7)]  **Resource use:**  Published guidelines, research studies, and expert opinion  **Unit costs:**  2001 Average Wholesale Prices  **Discount rate:**  3% for costs and health  **Currency (Price year):**  US dollar (2001) | **Deterministic sensitivity:**  Utility, costs, treatment efficacy and discount rate  **Probabilistic sensitivity:**  No  **Value of information:**  No | **Base-case:**  HRT for a 65-year-old woman with node (+) ER (+) disease is more cost-effective, $10,194/QALY, than CMF or AC chemotherapy.  **Probabilistic analysis:**  Not applicable.  **Value of information:**  Not applicable.  **Key drivers of relative cost-effectiveness:**  Discount rate |

Table S2. Full Data Extraction Table for [Naeim A. and Keeler E. B. (2005) [8]](#_ENREF_8)

| **Study Design** | **Study Characteristics** | **Data Sources** | **Analysis** | **Results** |
| --- | --- | --- | --- | --- |
| **Target population:**  65-year-old women to 75-and 85-year-old women with early-stage node (+) breast cancer  **Alternatives:**  Surgery with five treatment options were considered:  (a) Cyclophosphamide, methotrexate, and 5-flurouracil (CMF) × 6 chemotherapy,  (b) Adriamycin, cyclophosphamide (AC) × 4 chemotherapy,  (c) Tamoxifen hormone therapy (HRT)-5 years,  (d) Tamoxifen (HRT)-CMF,  (e) Tamoxifen (HRT)-AC.  **Country:**  USA | **Evaluation method:**  CEA  **Model type:**  Not stated  **Time horizon:**  10 years  **Perspective:**  health care provider  **Benefit measure:**  QALY  **Direct costs included:**  initial treatment  **Indirect costs included:**  Not applicable | **Effectiveness:**  Benefits of adjuvant therapy: meta-analyses conducted by the EBCTCG [[2-5](#_ENREF_2)] Transition probability: 1997 odds reduction of mortality data from the National Centre of Health Statistics. Economic data [[9](#_ENREF_9)]  **Health-related quality of life:**  previous reviews[[6](#_ENREF_6), [7](#_ENREF_7)]  **Resource use:**  Published guidelines, research studies, and expert opinion  **Unit costs:**  2001 Average Wholesale Prices (AWP)  **Discount rate:**  3% for costs and health  **Currency (Price year):**  US dollar (2001) | **Deterministic sensitivity:**  Utility, costs, treatment efficacy and discount rate  **Probabilistic sensitivity:**  No  **Value of information:**  No | **Base-case:**  HRT for a 65-year-old woman with node (+) ER (+) disease is more cost-effective, $6,520/QALY, than CMF or AC chemotherapy.  **Probabilistic analysis:**  Not applicable.  **Value of information:**  Not applicable.  **Key drivers of relative cost-effectiveness:**  Discount rate |

Table S3. Full Data Extraction Table for [Skedgel C., Rayson D.*et al* (2013) [10]](#_ENREF_10)

| **Study Design** | **Study Characteristics** | **Data Sources** | **Analysis** | **Results** |
| --- | --- | --- | --- | --- |
| **Target population:**  Women with T1bN0 breast cancer aged 40, 50, 60 and 70  **Alternatives:**  Four strategies:   1. no adjuvant chemotherapy or trastuzumab (baseline), 2. adjuvant chemotherapy alone, 3. adjuvant chemotherapy plus concurrent trastuzumab and 4. adjuvant chemotherapy plus sequential trastuzumab.   **Country:**  Canada | **Evaluation method:**  CEA  **Model type:**  Markov model  **Time horizon:**  Lifetime  **Perspective:**  Direct payer  **Benefit measure:**  QALY  **Direct costs included:**  HER-2/neu testing, acquisition costs, drug administration, supportive medications and cardiac monitoring  **Indirect costs included:**  Not applicable | **Effectiveness:**  Efficacy: clinical trials [[11-13](#_ENREF_11)]  Recurrences and adverse side-effects: previous literature [[11](#_ENREF_11), [13-16](#_ENREF_13)]  **Health-related quality of life:**  Cost-Effectiveness Analysis (CEA) Registry and previous literature [[17](#_ENREF_17)].  **Resource use:**  Previous literature [[11-16](#_ENREF_11)]  **Unit costs:**  Statistics Canada consumer price index, health and personal care component  **Discount rate:**  3% for costs and health  **Currency (Price year):**  Canadian dollars (2011) | **Deterministic sensitivity:**  Cost, trastuzumab duration of benefit, treatment effect, utility, and discount rate.  **Probabilistic sensitivity:**  Yes  **Value of information:**  No  [[12](#_ENREF_12)] | **Base-case:**  Strategy iii had a greater than 50% likelihood of meeting a $100 000 per QALY gained threshold at 10-year baseline recurrence rates more than 29%–35% in the ages 40, 50 and 60 cohorts.  **Probabilistic analysis:**  The minimum recurrence rates necessary to meet a $100 000 threshold were reduced to between 23% and 30% under strategy iii for the age 40, 50 and 60 cohorts and to 35% in the age 40 cohort under strategy iv.  **Value of information:**  Not applicable.  **Key drivers of relative cost-effectiveness:**  Chemotherapy without trastuzumab over the entire recurrence range tested |

Table S4. Full Data Extraction Table for [Sen S., Wang S. Y.*et al* (2014) [18]](#_ENREF_18)

| **Study Design** | **Study Characteristics** | **Data Sources** | **Analysis** | **Results** |
| --- | --- | --- | --- | --- |
| **Target population:**  Older women with early-stage breast cancer aged 70, 75, and 80 years  **Alternatives:**  Four strategies:   1. No Radiotherapy 2. External beam radiation therapy (EBRT) 3. Intensity modulated RT (IMRT)   **Country:**  USA | **Evaluation method:**  CEA  **Model type:**  Markov model  **Time horizon:**  Lifetime  **Perspective:**  Payer  **Benefit measure:**  QALY  **Direct costs included:**  Initial treatment, costs to Medicare (inpatient, outpatient facility, physician, home health, hospice, and Durable Medical Equipment claims)  **Indirect costs included:**  Not applicable | **Effectiveness:**  Transition probability: C9343 trial [[19](#_ENREF_19)];  Overall survival: The Surveillance, Epidemiology, and End Results (SEER)–Medicare database.  **Health-related quality of life:**  The literature [[20-22](#_ENREF_20)]  **Resource use:**  Observational data and literature [[23](#_ENREF_23)]  **Unit costs:**  The Surveillance, Epidemiology, and End Results (SEER)–Medicare database  **Discount rate:**  3% for costs and health  **Currency (Price year):**  US dollar (2012) | **Deterministic sensitivity:**  One-way sensitivity analysis: the cost of RT, utility of RT, treated-recurrence probability, metastasis probability, and cost of recurrence;  Two-way sensitivity analysis: The reduction in recurrence and improvement in age-specific QoL would need to be for the newer modalities to be cost-effective.  **Probabilistic sensitivity:**  Yes  **Value of information:**  No | **Base-case:**  The ICER for EBRT of $38300 per QALY. The ICER for IMRT were between $70200 per QALY and $79300 per QALY  **Probabilistic analysis:**  EBRT had a 54.6% probability of cost-effectiveness over no RT at a willingness-to-pay threshold of $100000 per QALY for women aged 70 years  **Value of information:**  Not applicable.  **Key drivers of relative cost-effectiveness:**  The utility benefit of RT |

Table S5. Full Data Extraction Table for [Ward M. C., Vicini F.*et al* (2020) [24]](#_ENREF_24)

| **Study Design** | **Study Characteristics** | **Data Sources** | **Analysis** | **Results** |
| --- | --- | --- | --- | --- |
| **Target population:**  Patients age 70 years or older with estrogen positive invasive breast cancer  **Alternatives:**  Three strategies:   1. an aromatase inhibitor (AI-alone) for 5 years, 2. a 5-fraction course of accelerated partial-breast irradiation using intensity-modulated radiation therapy (APBI-alone), 3. their combination.   **Country:**  USA | **Evaluation method:**  CEA  **Model type:**  Patient-level Markov microsimulation  **Time horizon:**  Lifetime  **Perspective:**  Societal  **Benefit measure:**  QALY  **Direct costs included:**  Treatment, imaging test and lab test, toxicity and salvage treatment  **Indirect costs included:**  Costs for the consult, simulation, treatment visits, and follow-up visit. | **Effectiveness:**  Transition probability: clinical trials[[25-32](#_ENREF_25)]  **Health-related quality of life:**  Literature [[33](#_ENREF_33)]  **Resource use:**  Hospital database and guidelines  **Unit costs:**  United States Bureau of Labor Statistics Consumer Price Index Inflation Calculator 2020  **Discount rate:**  3% for costs and health  **Currency (Price year):**  US dollars (2019) | **Deterministic sensitivity:**  One-way deterministic sensitivity analysis was performed on each parameter individually  **Probabilistic sensitivity:**  Yes  **Value of information:**  No | **Base-case:**  The strategy of AI-alone ($12,637) was cheaper than both APBI-alone ($13,799) and combination therapy ($18,012).  **Probabilistic analysis:**  AI-alone was cost-effective at $100,000/QALY in 50% of trials, APBI-alone in 28% and the combination in 22%.  **Value of information:**  Not applicable  **Key drivers of relative cost-effectiveness:**  Not reported |

Table S6. Full Data Extraction Table for [Ward M. C., Vicini F. (2019) [30]](#_ENREF_30)

| **Study Design** | **Study Characteristics** | **Data Sources** | **Analysis** | **Results** |
| --- | --- | --- | --- | --- |
| **Target population:**  Patient aged 70 years or older with early-stage breast cancer  **Alternatives:**  Two adjuvant therapy strategies were considered:   1. AI without radiation therapy (AI-alone, “standard”); 2. Radiation therapy without AI (RT-alone, “experimental”).   **Country:**  USA | **Evaluation method:**  CEA  **Model type:**  Markov microsimulation model  **Time horizon:**  Lifetime  **Perspective:**  Societal  **Benefit measure:**  QALY  **Direct costs included:**  Treatment, imaging test and lab test, toxicity and salvage treatment  **Indirect costs included:**  Costs for the consult, simulation, treatment visits, and follow-up visit. | **Effectiveness:**  Transition probability: clinical trials[[25-32](#_ENREF_25)]  **Health-related quality of life:**  Literature [[33](#_ENREF_33)]  **Resource use:**  Hospital database and guidelines  **Unit costs:**  United States Bureau of Labor Statistics Consumer Price Index Inflation Calculator 2019  **Discount rate:**  3% for costs and health  **Currency (Price year):**  US dollars (2018) | **Deterministic sensitivity:**  One-way deterministic sensitivity analysis was performed on each parameter individually  **Probabilistic sensitivity:**  Yes  **Value of information:**  No | **Base-case:**  The overall ICER of the base case for RT alone compared with AI alone was $210,101 per QALY on average.  **Probabilistic analysis:**  In 62% of trials, the AI-only strategy was more cost-effective than RT only at the $100,000-per-QALY threshold.  A display the acceptability curve, with AI alone as the preferred strategy for all willingness-to-pay levels less than approximately $200,000 per QALY.  **Value of information:**  Not applicable  **Key drivers of relative cost-effectiveness:**  Cardiac comorbidities were a more significant driver of mortality than radiation therapy |

Table S7. Full Data Extraction Table for [Desch C. E., Hillner B. E.*et al* (1993) [34]](#_ENREF_34)

| **Study Design** | **Study Characteristics** | **Data Sources** | **Analysis** | **Results** |
| --- | --- | --- | --- | --- |
| **Target population:**  Postmenopausal women from ages 60 to 80 years with a diagnosis of primary breast cancer  **Alternatives:**  Adjuvant chemotherapy in elderly women with breast cancer  **Country:**  USA | **Evaluation method:**  CUA and CEA  **Model type:**  Markov model  **Time horizon:**  Lifetime  **Perspective:**  Societal  **Benefit measure:**  QALY  **Direct costs included:**  Costs to the health service of chemotherapy, major and minor toxicity  **Indirect costs included:**  Not considered | **Effectiveness:**  Transition probability: Clinical trials [[35](#_ENREF_35), [36](#_ENREF_36)]  **Health-related quality of life:**  Literature [[37](#_ENREF_37)]  **Resource use:**  Costs and quantities of resources were not separately identified. The costs in the last year of life were an estimate based on two published estimates.  **Unit costs:**  Charges in 1989 at the Medical College of Virginia and estimates from Medicare data  **Discount rate:**  5% for costs and health  **Currency (Price year):**  US dollars (1990) | **Deterministic sensitivity:**  A set of one-way and multi-way sensitivity analyses were performed on the parameters of the model.  **Probabilistic sensitivity:**  No  **Value of information:**  No | **Base-case:**  The costs per QALY were  $28,200 (aged 60), $31,300 (aged 65), $36,300 (aged 70), $44,400 (aged 75) and $57,100 (aged 80)  Using active life expectancy, the costs per QALY of adjuvant chemotherapy in elderly women with breast cancer increased to $59,300 (aged 65), $75,000 (aged 70), $96,000 (aged 75) and $212,500 (aged 80).  **Probabilistic analysis:**  Not applicable  **Value of information:**  Not applicable  **Key drivers of relative cost-effectiveness:**  Not reported |

# Supplementary Appendix 4. Details of Input Parameters

Table S4. Sources of evidence to estimate health-related quality of life

| **Author**  **(year)** | **Age of target population** | **Health state** | **Utility value** | **Instrument** | **Data source** | **Sample size (n)** | **Mean age of estimation sample** | **Method of age adjustment** |
| --- | --- | --- | --- | --- | --- | --- | --- | --- |
| [Naeim A. and Keeler E. B. (2005) [1]](#_ENREF_1) | 65-85 years | **Disease free**  Baseline  **Progression**:  Baseline for hormone therapy  Baseline for minor toxicity with chemotherapy  Baseline for major toxicity with chemotherapy | 1.0  0.99  0.9  0.8 | Not reported | Expert elicitation [[6](#_ENREF_6), [7](#_ENREF_7)] | 150 | Not reported | No |
| [Naeim A. and Keeler E. B. (2005) [8]](#_ENREF_8) | 65-85 years | **Disease free**  Baseline  **Progression**:  Baseline for hormone therapy  Baseline for minor toxicity with chemotherapy  Baseline for major toxicity with chemotherapy | 1.0  0.99  0.9  0.8 | Not reported | Expert elicitation [[6](#_ENREF_6), [7](#_ENREF_7)] | 150 | Not reported | No |
| [Skedgel C., Rayson D. (2013) [10]](#_ENREF_10) | 40 years  50 years  60 years  70 years | **Disease** **free**:  Disease-free baseline, 70–79  Disease-free baseline, 80+  **Progression**:  First local recurrence  Second local recurrence  Well after relapse  Distant recurrence  **Side** **effect**  Congestive heart failure  Febrile neutropenia  AML/MDS  Nausea/vomiting | 0.81  0.78  0.70  0.50  0.90  0.60  0.87  0.47  0.26  0.85 | EQ-5D-3L for baseline value | Data derived from previous literature [[17](#_ENREF_17)] for the baseline utility values | 2981 [[17](#_ENREF_17)] | 74 years [[17](#_ENREF_17)] | Partial adjustment:  Age-dependent baseline values, and fixed progression state values |
|  |  |  |  | Not reported for health-related Quality of Life | Health-related Quality of Life was identified from the Cost-Effectiveness Analysis Registry | Not reported | Not reported |  |
| [Sen S., Wang S. Y. (2014) [18]](#_ENREF_18) | 70, 75, and 80 years | **Utilities according to treatment and recurrence status:**  Conservative surgery and radiation therapy with no recurrence  Conservative surgery and radiation therapy with isolated local recurrence  Conservative surgery alone with no recurrence  Conservative surgery alone with isolated local recurrence  Distant metastases  **Utility modifier according to age**  70–74 y  75–79 y  80–84 y  >85 y | 0.92  0.82  0.88  0.81  0.71  0.716  0.675  0.623  0.59 | 1. EQ-5D [[20](#_ENREF_20)]  2. Standard Gambles [[22](#_ENREF_22)] | 1.Recurrence value elicited from previous literature [[20](#_ENREF_20)]  2.value derived from previous literature [[22](#_ENREF_22)] | 1.97 [[20](#_ENREF_20)]  Not reported  2. Not reported using national censor data source [[22](#_ENREF_22)] | Not reported [[20](#_ENREF_20)]  The age-specific group reported from 30 to 85 with 5-year interval-group [[22](#_ENREF_22)] | Age-dependent baseline values, and health-state utilities by multiplying the standard gamble utilities by the mean age-specific utility |
| [Ward M. C., Vicini F. (2020) [24]](#_ENREF_24) | 70 years or older | **Disease free**  Baseline  **Progression**  *Distant Metastasis  *Second Malignancy: Radiation Induced  *Salvage Mastectomy  *Salvage Axillary Dissection After | 0.84 [[33](#_ENREF_33)]  0.22 [[38](#_ENREF_38), [39](#_ENREF_39)]  0.18 [[40-42](#_ENREF_40)]  0.16 [[38](#_ENREF_38), [43](#_ENREF_43)]  0.16 [[44-46](#_ENREF_44)] | EQ-5D | Values derived from a cross-sectional U.S. population survey 2005 [[33](#_ENREF_33)]  Disutility values from previous literature [[47](#_ENREF_47)] | 965 of a sub-cohort for the patients aged 65-74 years | 65-74 years [[33](#_ENREF_33)] | Age-dependent baseline values, and health-state utilities with an additive utility decrement |
|  |  | Axillary Recurrence  **Side effect**  *Fracture  *Second malignancy: Endometrial cancer  *Salvage Lumpectomy with Radiation  *Treatment of Contralateral Cancer  *Cardiac Adverse Event (MI)  *DVT  *Acute Radiation Dermatitis, Grade 3  *Hot Flashes  *Arthralgia  *Late Radiation-induced Fibrosis | 0.13 [[40](#_ENREF_40), [48](#_ENREF_48)]  0.12 [[49-52](#_ENREF_49)]  0.10 [[43](#_ENREF_43)]  0.08 [[43](#_ENREF_43)]  0.07 [[53](#_ENREF_53), [54](#_ENREF_54)]  0.05 [[55](#_ENREF_55), [56](#_ENREF_56)]  0.02[[57](#_ENREF_57)]  0.01  0.01  0.01[[57](#_ENREF_57)] |  |  |  |  |  |
| [Ward M. C., Vicini F. (2019) [30]](#_ENREF_30) | 70 years or older | **Disease free**  Baseline  **Progression**  *Distant Metastasis  *Second Malignancy: Radiation Induced  *Salvage Mastectomy  *Salvage Axillary Dissection After | 0.84 [[33](#_ENREF_33)]  0.22 [[38](#_ENREF_38), [39](#_ENREF_39)]  0.18 [[40-42](#_ENREF_40)]  0.16 [[38](#_ENREF_38), [43](#_ENREF_43)]  0.16 [[44-46](#_ENREF_44)] | EQ-5D | Values derived from a cross-sectional U.S. population survey 2005 [[33](#_ENREF_33)]  Disutility values from previous literature [[47](#_ENREF_47)] | 965 of a sub-cohort for the patients aged 65-74 years | 65-74 years[[33](#_ENREF_33)] | Age-dependent baseline values, and health-state utilities with an additive utility decrement |
|  |  | Axillary Recurrence  **Side effect**  *Fracture  *Second malignancy: Endometrial cancer  *Salvage Lumpectomy with Radiation  *Treatment of Contralateral Cancer  *Cardiac Adverse Event (MI)  *DVT  *Acute Radiation Dermatitis, Grade 3  *Hot Flashes  *Arthralgia  *Late Radiation-induced Fibrosis | 0.13 [[40](#_ENREF_40), [48](#_ENREF_48)]  0.12 [[49-52](#_ENREF_49)]  0.10 [[43](#_ENREF_43)]  0.08 [[43](#_ENREF_43)]  0.07 [[53](#_ENREF_53), [54](#_ENREF_54)]  0.05 [[55](#_ENREF_55), [56](#_ENREF_56)]  0.02[[57](#_ENREF_57)]  0.01  0.01  0.01[[57](#_ENREF_57)] |  |  |  |  |  |
| [Desch C. E., Hillner B. E. (1993) [34]](#_ENREF_34) | 60 years  65 years  70 years  75 years  80 years | **Disease free**  Well  **Progression**:  First recurrence  **Side effect**  Minor toxicity with chemotherapy  Major toxicity with chemotherapy | 1.0  0.7  0.9  0.8 | Not reported | Assumption without justification | NA | NA | NA |

(Note) DVT: Deep vein thrombosis; AML/MDS, acute myeloid leukaemia and/or myelodysplastic syndrome; MI: myocardial infarction; * disutility used in the study.

Table 5. Sources of evidence to estimate transition probabilities

| **Economic Evaluation** | | **Source of Evidence to Estimate Natural History of Disease** | | | | | |
| --- | --- | --- | --- | --- | --- | --- | --- |
| **Author**  **(Year)** | **Age of Target Population** | **Probability** | **Value** | **Data Source** | **Sample Size**  **(n)** | **Mean Age of Estimation Sample** |  |
| [Naeim A. and Keeler E. B. (2005) [1]](#_ENREF_1) | 65-85 years | * odds reduction of mortality for CMF × 6  * odds reduction of mortality for AC ×4  * odds reduction of mortality for HRT × 5 year  * odds reduction of mortality for HRT× 5 year + CMF  * odds reduction of mortality for HRT× 5 year +AC | 0.02  0.033  0.25  0.25-0.36  0.27-0.38 | United States life tables, 1997 [[9](#_ENREF_9)];  benefits of adjuvant therapy: meta-analyses conducted by the EBCTCG [[2-5](#_ENREF_2)] | US population | Age-specific mortality from 0 to 100 years |  |
| [Naeim A. and Keeler E. B. (2005) [8]](#_ENREF_8) | 65-85 years | * odds reduction of mortality for CMF × 6  * odds reduction of mortality for AC ×4  * odds reduction of mortality for HRT × 5 year  * odds reduction of mortality for HRT× 5 year + CMF  * odds reduction of mortality for HRT× 5 year +AC | 0.02  0.033  0.25  0.25-0.36  0.27-0.38 | United States life tables, 1997 [[9](#_ENREF_9)];  benefits of adjuvant therapy: meta-analyses conducted by the EBCTCG [[2-5](#_ENREF_2)] | US population | Age-specific mortality from 0 to 100 years |  |
| [Skedgel C., Rayson D. (2013) [10]](#_ENREF_10) | 40 years  50 years  60 years  70 years | Proportion local recurrence/recurrence  ‘Instant’ conversion from local to distant  Rate of nausea\|vomiting (grades 3 + 4)  Rate of febrile neutropenia  Rate of CHF  Relative mortality risk\|CHF  Rate of AML/MDS  Relative mortality rate\|AML/MDS  Relative risk of cardiotoxicity\|conTZ  Relative risk of cardiotoxicity\|seqTZ | 25%  20%  2.96%  4.94%  0.02%  2.00  0.39%  2.00  115.68  90.38 | Recurrences from clinical trials [[11](#_ENREF_11), [13](#_ENREF_13)] and meta-analysis [[12](#_ENREF_12)]; and adverse side-effects from previous literature [[11](#_ENREF_11), [13-16](#_ENREF_13)] | 1703[[11](#_ENREF_11)]  1944 [[13](#_ENREF_13)]  Not reported [[12](#_ENREF_12)] | Patients aged >70 years account for 16% [[11](#_ENREF_11)]  Patients aged >60 years account for 16.3% [[13](#_ENREF_13)]  50-69 years [[12](#_ENREF_12)] |  |
| [Sen S., Wang S. Y. (2014) [18]](#_ENREF_18) | 70, 75, and 80 years | Transition probability: disease-free to recurrence no RT  Transition probability: disease-free to recurrence + RT  Transition probability: recurrence to metastasis  Transition probability: metastasis to death | 0.01 12 months  0.18 12 months  0.005 12 months  0.210-0.238 12 months | Clinical trial [[25](#_ENREF_25)] | 636 | > 70years |  |
| [Ward M. C., Vicini F. (2020) [24]](#_ENREF_24) | 70 years or older | # Cumulative incidence for ipsilateral breast tumours recurrence  # Cumulative incidence for distant metastasis  # Cumulative incidence for contralateral breast cancer  # Cumulative incidence for overall survival  # Cumulative incidence for death from 2^nd^ cancer  # Cumulative incidence for Osteopenia requiring bisphosphonate  # Cumulative incidence for bone fracture  # Cumulative incidence for deep vein thrombosis  # Cumulative incidence for fibrosis/soft-tissue necrosis  # Cumulative incidence for hot flashes  # Cumulative incidence for arthralgia  # Cumulative incidence for radiation dermatitis, acute grade 3 | APBI: 2.1%  AI: 3.9%  APBI+AI: 1.1%  APBI: 2.1%  AI: 1.8%  APBI+AI: 1.8%  APBI: 3.0%  AI: 0.8%  APBI+AI: 0.8%  APBI: 80.2%  AI: 80.4%  APBI+AI: 80.4%  APBI: 0.28%  AI: 0.08%  APBI+AI: 0.11%  APBI: 4.7%  AI: 16.3%  APBI+AI: 16.3%  APBI: 13.0%  AI: 15.1%  APBI+AI: 15.1%  APBI: 0%  AI: 1.60%  APBI+AI: 1.59%  APBI: 0.7%  AI: 0%  APBI+AI: 0.7%  APBI: 16.0%  AI: 34.9%  APBI+AI: 34.9%  APBI: 0%  AI: 6.2%  APBI+AI: 6.2%  APBI: 0%  AI: 0%  APBI+AI: 0.5% | Clinical trials[[25-32](#_ENREF_25)] | 636 [[25](#_ENREF_25)]  1326 [[26](#_ENREF_26)]  869 [[27](#_ENREF_27)]  1135 [[28](#_ENREF_28)]  1009 [[29](#_ENREF_29)] | > 70years [[25](#_ENREF_25)]  >65 years [[26](#_ENREF_26)]  65.7 years [[27](#_ENREF_27)]  57 years [[28](#_ENREF_28)]  Not reported [[29](#_ENREF_29)] |  |
| [Ward M. C., Vicini F. (2019) [30]](#_ENREF_30) | 70 years or older | # Cumulative incidence for ipsilateral breast tumours recurrence  # Cumulative incidence for distant metastasis  # Cumulative incidence for contralateral breast cancer  # Cumulative incidence for overall survival  # Cumulative incidence for death from 2^nd^ cancer  # Cumulative incidence for Osteopenia requiring bisphosphonate  # Cumulative incidence for bone fracture  # Cumulative incidence for deep vein thrombosis  # Cumulative incidence for fibrosis/soft-tissue necrosis  # Cumulative incidence for hot flashes  # Cumulative incidence for arthralgia  # Cumulative incidence for radiation dermatitis, acute grade 3 | APBI: 2.1%  AI: 3.9%  APBI+AI: 1.1%  APBI: 2.1%  AI: 1.8%  APBI+AI: 1.8%  APBI: 3.0%  AI: 0.8%  APBI+AI: 0.8%  APBI: 80.2%  AI: 80.4%  APBI+AI: 80.4%  APBI: 0.28%  AI: 0.08%  APBI+AI: 0.11%  APBI: 4.7%  AI: 16.3%  APBI+AI: 16.3%  APBI: 13.0%  AI: 15.1%  APBI+AI: 15.1%  APBI: 0%  AI: 1.60%  APBI+AI: 1.59%  APBI: 0.7%  AI: 0%  APBI+AI: 0.7%  APBI: 16.0%  AI: 34.9%  APBI+AI: 34.9%  APBI: 0%  AI: 6.2%  APBI+AI: 6.2%  APBI: 0%  AI: 0%  APBI+AI: 0.5% | Clinical trials[[25-32](#_ENREF_25)] | 636 [[25](#_ENREF_25)]  1326 [[26](#_ENREF_26)]  869 [[27](#_ENREF_27)]  1135 [[28](#_ENREF_28)]  1009 [[29](#_ENREF_29)] | > 70years [[25](#_ENREF_25)]  >65 years [[26](#_ENREF_26)]  65.7 years [[27](#_ENREF_27)]  57 years [[28](#_ENREF_28)]  Not reported [[29](#_ENREF_29)] |  |
| [Desch C. E., Hillner B. E. (1993) [34]](#_ENREF_34) | 60 years  65 years  70 years  75 years  80 years | First recurrence  Relative reduction in breast cancer recurrence with chemotherapy | 5  20 | Clinical trials [[35](#_ENREF_35), [36](#_ENREF_36)] | 679 [[35](#_ENREF_35)]  524 [[36](#_ENREF_36)] | 48 years [[35](#_ENREF_35)]  Not reported [[36](#_ENREF_36)] |  |

Note: *Odds reduction used from 10-year mortality; # 5 years cumulative incidence

seqTZ, Sequential trastuzumab; conTZ, concurrent trastuzumab; AML/MDS, acute myeloid leukaemia and/or myelodysplastic syndrome; CHF, chemotherapy-related congestive heart failure; AI: Aromatase inhibitor; Accelerated partial-breast irradiation: APBI

Table 6. Sources of Evidence to Estimate Resource use

| **Economic Evaluation** | | **Source of Evidence to Estimate Resource Use** | | | | |
| --- | --- | --- | --- | --- | --- | --- |
| **Author**  **(year)** | **Age of target population** | **Health state** | **Resource estimate** | **Data source** | **Sample size (n)** | **Mean age of estimation sample** |
| [Naeim A. and Keeler E. B. (2005) [1]](#_ENREF_1) | 65-85 years | CMF × 6  AC ×4  HRT × 5 years  HRT× 5 years + CMF  HRT× 5 years +AC | $4568 (AWP) $2833 (PHS)  $5965 (AWP) $2318 (PHS)  $6320 (AWP) $3350 (PHS)  $10,923 (AWP) $6201 (PHS)  $12,320 (AWP) $5686 (PHS) | Published guidelines, research studies, and expert opinion | Not reported | Not reported |
| [Naeim A. and Keeler E. B. (2005) [8]](#_ENREF_8) | 65-85 years | CMF × 6  AC ×4  HRT × 5 year  HRT× 5 year + CMF  HRT× 5 year +AC | $4568 (AWP) $2833 (PHS)  $5965 (AWP) $2318 (PHS)  $6320 (AWP) $3350 (PHS)  $10,923 (AWP) $6201 (PHS)  $12,320 (AWP) $5686 (PHS) | Published guidelines, research studies, and expert opinion | Not reported | Not reported |
| [Skedgel C., Rayson D. (2013) [10]](#_ENREF_10) | 40 years  50 years  60 years  70 years | TC course  FEC-D course  12 months adjuvant trastuzumab, per case  Local recurrence, per case  Distant recurrence, per case  Post-recurrence follow-up, per month  Febrile neutropenia, per case  AML/MDS, per month  Chemo-related CHF, per month  Chemo-related nausea and vomiting, per case  Trastuzumab-related cardiotoxicity, per month  Palliative trastuzumab, per case | $4345  $9055  $55,617  $12,522  $38,088  $45  $18,685  $5964  $1715  $22  $669  $31,241 | Previous literature [[11-16](#_ENREF_11)] | Not reported | Not reported |
| [Sen S., Wang S. Y. (2014) [18]](#_ENREF_18) | 70, 75, and 80 years | No RT  EBRT  IMRT  Brachytherapy  Recurrence, mastectomy  Metastatic care  Continued phase  Death, last year of life | $5593  $15396  $23605  $23628  $6250  $37771  $284 (2–4 y); *$212 (after year 4)  $44732 | SEER-Medicare  *Previous literature [[23](#_ENREF_23)] | Not reported | 70-74 years; 75-79 years; 80-94 years |
| [Ward M. C., Vicini F. (2020) [24]](#_ENREF_24) | 70 years or older | Radiation Therapy  Anastrozole (per year)  Indirect costs of RT  Indirect costs of Endocrine Therapy (Annual)  Salvage Mastectomy  Salvage Lumpectomy or Axillary Dissection  Metastatic Disease (per year) | $5,590  $989  $275  $150  $13,378  $2,632  $23,460 | ASCO and National Cancer Centers Network (NCCN) guidelines, all costs were adjusted to 2019 dollars using the US Bureau of Labor Statistics overall Consumer Price Index inflation | Not reported | Not reported |
| [Ward M. C., Vicini F. (2019) [30]](#_ENREF_30) | 70 years or older | Radiation Therapy  Anastrozole (per year)  Indirect costs of RT  Indirect costs of Endocrine Therapy (Annual)  Salvage Mastectomy  Salvage Lumpectomy or Axillary Dissection  Metastatic Disease (per year) | $7476  $970  $595  $147  $13116  $2580  $23000 | ASCO and National Cancer Centers Network (NCCN) guidelines, all costs were adjusted to 2018 dollars using the US Bureau of Labor Statistics overall Consumer Price Index inflation calculator | Not reported | Not reported |
| [Desch C. E., Hillner B. E. (1993) [34]](#_ENREF_34) | 60 years  65 years  70 years  75 years  80 years | Chemotherapy, if given  Minor toxicity  Major toxicity | $6000  $1500  $10000 | Previous literature [[37](#_ENREF_37)]  Medical College of Virginia and estimates from Medicare data (1989) | Not reported | Not reported |

Note: AC: adriamycin, cyclophosphamide; CMF: cyclophosphamide, methotrexate, and 5-fluorouracil; HRT: tamoxifen hormone therapy; AWP: Average Wholesale Prices; PHS: Public Health Service; EBRT: external beam radiation therapy; RT: radiation therapy; IMRT: intensity-modulated RT

# Reference

1. Naeim, A. and Keeler, E.B., Is adjuvant therapy for older patients with node (-) early breast cancer cost-effective? Crit Rev Oncol Hematol, 2005. **53**(1): p. 81-9.

2. Peto, R.J.H.R.i.P., Effects of adjuvant tamoxifen and of cytotoxic therapy on mortality in early breast cancer. An overview of 61 randomised trials among 28,896 women. 1989. **32**(Suppl. 1): p. 165-165.

3. Lancet, E.B.C.T.C.G.J.T., Systemic treatment of early breast cancer by hormonal, cytotoxic, or immune therapy: 133 randomised trials involving 31 000 recurrences and 24 000 deaths among 75 000 women. 1992. **339**(8784): p. 1-15.

4. Lancet, E.B.C.T.C.G.J.T., Tamoxifen for early breast cancer: an overview of the randomised trials. 1998. **351**(9114): p. 1451-1467.

5. Lancet, E.B.C.T.C.G.J.T., Polychemotherapy for early breast cancer: an overview of the randomised trials. 1998. **352**(9132): p. 930-942.

6. Kattlove, H., Liberati, A., Keeler, E.*, et al.*, Benefits and costs of screening and treatment for early breast cancer. Development of a basic benefit package. JAMA, 1995. **273**(2): p. 142-8.

7. Malin, J. and Keeler, E.J.R., Los Angeles, Cost-effectiveness and health benefit of breast cancer in women with early breast cancer. 2000.

8. Naeim, A. and Keeler, E.B., Is adjuvant therapy for older patients with node (+) early breast cancer cost-effective? Breast Cancer Res Treat, 2005. **94**(2): p. 95-103.

9. Anderson, R.N., United States life tables, 1997. Natl Vital Stat Rep, 1999. **47**(28): p. 1-37.

10. Skedgel, C., Rayson, D., and Younis, T., Is adjuvant trastuzumab a cost-effective therapy for HER-2/neu-positive T1bN0 breast cancer? Ann Oncol, 2013. **24**(7): p. 1834-1840.

11. Smith, I., Procter, M., Gelber, R.D.*, et al.*, 2-year follow-up of trastuzumab after adjuvant chemotherapy in HER2-positive breast cancer: a randomised controlled trial. Lancet, 2007. **369**(9555): p. 29-36.

12. Lancet, E.B.C.T.C.G.J.T., Effects of chemotherapy and hormonal therapy for early breast cancer on recurrence and 15-year survival: an overview of the randomised trials. 2005. **365**(9472): p. 1687-1717.

13. Perez, E.A., Romond, E.H., Suman, V.J.*, et al.*, Four-year follow-up of trastuzumab plus adjuvant chemotherapy for operable human epidermal growth factor receptor 2-positive breast cancer: joint analysis of data from NCCTG N9831 and NSABP B-31. J Clin Oncol, 2011. **29**(25): p. 3366-73.

14. Skedgel, C., Rayson, D., and Younis, T., The cost-utility of sequential adjuvant trastuzumab in women with Her2/Neu-positive breast cancer: an analysis based on updated results from the HERA Trial. Value Health, 2009. **12**(5): p. 641-8.

15. Younis, T., Rayson, D., and Skedgel, C.J.C.O., The cost–utility of adjuvant chemotherapy using docetaxel and cyclophosphamide compared with doxorubicin and cyclophosphamide in breast cancer. 2011. **18**(6): p. e288.

16. Younis, T., Rayson, D., Sellon, M.*, et al.*, Adjuvant chemotherapy for breast cancer: a cost-utility analysis of FEC-D vs. FEC 100. Breast Cancer Res Treat, 2008. **111**(2): p. 261-7.

17. Sullivan, P.W. and Ghushchyan, V., Preference-Based EQ-5D index scores for chronic conditions in the United States. Med Decis Making, 2006. **26**(4): p. 410-20.

18. Sen, S., Wang, S.Y., Soulos, P.R.*, et al.*, Examining the cost-effectiveness of radiation therapy among older women with favorable-risk breast cancer. J Natl Cancer Inst, 2014. **106**(3): p. dju008.

19. Palta, M., Palta, P., Bhavsar, N.A.*, et al.*, The use of adjuvant radiotherapy in elderly patients with early‐stage breast cancer: Changes in practice patterns after publication of Cancer and Leukemia Group B 9343. 2015. **121**(2): p. 188-193.

20. Hayman, J.A., Fairclough, D.L., Harris, J.R.*, et al.*, Patient preferences concerning the trade-off between the risks and benefits of routine radiation therapy after conservative surgery for early-stage breast cancer. 1997. **15**(3): p. 1252-1260.

21. Folland, S., Goodman, A.C., and Stano, M., The Economics of Health and Health Care: Pearson New International Edition. 2016: Routledge.

22. Stout, N.K., Rosenberg, M.A., Trentham-Dietz, A.*, et al.*, Retrospective cost-effectiveness analysis of screening mammography. J Natl Cancer Inst, 2006. **98**(11): p. 774-82.

23. Rao, S., Kubisiak, J., and Gilden, D., Cost of illness associated with metastatic breast cancer. Breast Cancer Res Treat, 2004. **83**(1): p. 25-32.

24. Ward, M.C., Vicini, F., Al-Hilli, Z.*, et al.*, Cost-effectiveness analysis of endocrine therapy alone versus partial-breast irradiation alone versus combined treatment for low-risk hormone-positive early-stage breast cancer in women aged 70 years or older. Breast Cancer Res Treat, 2020. **182**(2): p. 355-365.

25. Hughes, K.S., Schnaper, L.A., Bellon, J.R.*, et al.*, Lumpectomy plus tamoxifen with or without irradiation in women age 70 years or older with early breast cancer: long-term follow-up of CALGB 9343. 2013. **31**(19): p. 2382.

26. Kunkler, I.H., Williams, L.J., Jack, W.J.*, et al.*, Breast-conserving surgery with or without irradiation in women aged 65 years or older with early breast cancer (PRIME II): a randomised controlled trial. Lancet Oncol, 2015. **16**(3): p. 266-73.

27. Potter, R., Gnant, M., Kwasny, W.*, et al.*, Lumpectomy plus tamoxifen or anastrozole with or without whole breast irradiation in women with favorable early breast cancer. Int J Radiat Oncol Biol Phys, 2007. **68**(2): p. 334-40.

28. Blamey, R.W., Bates, T., Chetty, U.*, et al.*, Radiotherapy or tamoxifen after conserving surgery for breast cancers of excellent prognosis: British Association of Surgical Oncology (BASO) II trial. Eur J Cancer, 2013. **49**(10): p. 2294-302.

29. Fisher, B., Bryant, J., Dignam, J.J.*, et al.*, Tamoxifen, radiation therapy, or both for prevention of ipsilateral breast tumor recurrence after lumpectomy in women with invasive breast cancers of one centimeter or less. J Clin Oncol, 2002. **20**(20): p. 4141-9.

30. Ward, M.C., Vicini, F., Chadha, M.*, et al.*, Radiation Therapy Without Hormone Therapy for Women Age 70 or Above with Low-Risk Early Breast Cancer: A Microsimulation. Int J Radiat Oncol Biol Phys, 2019. **105**(2): p. 296-306.

31. Group., E.B.C.T.C., Aromatase inhibitors versus tamoxifen in early breast cancer: patient-level meta-analysis of the randomised trials. The Lancet, 2015. **386**(10001): p. 1341-1352.

32. Early Breast Cancer Trialists' Collaborative, G., Relevance of breast cancer hormone receptors and other factors to the efficacy of adjuvant tamoxifen: patient-level meta-analysis of randomised trials. The Lancet, 2011. **378**(9793): p. 771-784.

33. Fryback, D.G., Dunham, N.C., Palta, M.*, et al.*, US norms for six generic health-related quality-of-life indexes from the National Health Measurement study. Med Care, 2007. **45**(12): p. 1162-70.

34. Desch, C.E., Hillner, B.E., Smith, T.J.*, et al.*, Should the elderly receive chemotherapy for node-negative breast cancer? A cost-effectiveness analysis examining total and active life-expectancy outcomes. J Clin Oncol, 1993. **11**(4): p. 777-82.

35. Fisher, B., Redmond, C., Dimitrov, N.V.*, et al.*, A randomized clinical trial evaluating sequential methotrexate and fluorouracil in the treatment of patients with node-negative breast cancer who have estrogen-receptor-negative tumors. N Engl J Med, 1989. **320**(8): p. 473-8.

36. Rosen, P.R., Groshen, S., Saigo, P.E.*, et al.*, A long-term follow-up study of survival in stage I (T1N0M0) and stage II (T1N1M0) breast carcinoma. J Clin Oncol, 1989. **7**(3): p. 355-66.

37. Hillner, B.E. and Smith, T.J., Efficacy and cost effectiveness of adjuvant chemotherapy in women with node-negative breast cancer. A decision-analysis model. N Engl J Med, 1991. **324**(3): p. 160-8.

38. Sen, S., Wang, S.-Y., Soulos, P.R.*, et al.*, Examining the cost-effectiveness of radiation therapy among older women with favorable-risk breast cancer. 2014. **106**(3).

39. Hannouf, M., Winquist, E., Mahmud, S.*, et al.*, Cost-effectiveness of using a gene expression profiling test to aid in identifying the primary tumour in patients with cancer of unknown primary. 2017. **17**(3): p. 286-300.

40. Suh, W.W., Hillner, B.E., Pierce, L.J.*, et al.*, Cost-effectiveness of radiation therapy following conservative surgery for ductal carcinoma in situ of the breast. Int J Radiat Oncol Biol Phys, 2005. **61**(4): p. 1054-61.

41. Djalalov, S., Beca, J., Amir, E.*, et al.*, Economic evaluation of hormonal therapies for postmenopausal women with estrogen receptor-positive early breast cancer in Canada. Curr Oncol, 2015. **22**(2): p. 84-96.

42. Soini, E.J.O., Garcia San Andres, B., and Joensuu, T., Trabectedin in the treatment of metastatic soft tissue sarcoma: cost-effectiveness, cost-utility and value of information. Ann Oncol, 2011. **22**(1): p. 215-223.

43. Verry, H., Lord, S.J., Martin, A.*, et al.*, Effectiveness and cost-effectiveness of sentinel lymph node biopsy compared with axillary node dissection in patients with early-stage breast cancer: a decision model analysis. Br J Cancer, 2012. **106**(6): p. 1045-52.

44. Songtish, D., Praditsitthikorn, N., and Teerawattananon, Y., A Cost-Utility Analysis Comparing Standard Axillary Lymph Node Dissection with Sentinel Lymph Node Biopsy in Patients with Early Stage Breast Cancer in Thailand. Value Health Reg Issues, 2014. **3**: p. 59-66.

45. Wong, S.L., Abell, T.D., Chao, C.*, et al.*, Optimal use of sentinel lymph node biopsy versus axillary lymph node dissection in patients with breast carcinoma: a decision analysis. 2002. **95**(3): p. 478-487.

46. Kwon, J.W., Park, H.Y., Kim, Y.J.*, et al.*, Cost-effectiveness of Pharmaceutical Interventions to Prevent Osteoporotic Fractures in Postmenopausal Women with Osteopenia. J Bone Metab, 2016. **23**(2): p. 63-77.

47. Ito, K., Blinder, V.S., and Elkin, E.B. Cost effectiveness of fracture prevention in postmenopausal women who receive aromatase inhibitors for early breast cancer. in *Presented at the*. 2012. Citeseer.

48. Green, L.E., Dinh, T.A., Hinds, D.A.*, et al.*, Economic evaluation of using a genetic test to direct breast cancer chemoprevention in white women with a previous breast biopsy. 2014. **12**(2): p. 203-217.

49. Wang, G., Kuppermann, M., Kim, B.*, et al.*, Influence of patient preferences on the cost-effectiveness of screening for lynch syndrome. 2012. **8**(3S): p. e24s-e30s.

50. Eckermann, S.D., Martin, A.J., Stockler, M.R.*, et al.*, The benefits and costs of tamoxifen for breast cancer prevention. Aust N Z J Public Health, 2003. **27**(1): p. 34-40.

51. Jiang, M. and You, J.H., Cost-effectiveness analysis of personalized antiplatelet therapy in patients with acute coronary syndrome. Pharmacogenomics, 2016. **17**(7): p. 701-13.

52. Lewis, E.F., Li, Y., Pfeffer, M.A.*, et al.*, Impact of cardiovascular events on change in quality of life and utilities in patients after myocardial infarction: a VALIANT study (valsartan in acute myocardial infarction). JACC Heart Fail, 2014. **2**(2): p. 159-65.

53. Lefebvre, P., Coleman, C.I., Bookhart, B.K.*, et al.*, Cost-effectiveness of rivaroxaban compared with enoxaparin plus a vitamin K antagonist for the treatment of venous thromboembolism. J Med Econ, 2014. **17**(1): p. 52-64.

54. Hogg, K., Kimpton, M., Carrier, M.*, et al.*, Estimating quality of life in acute venous thrombosis. JAMA Intern Med, 2013. **173**(12): p. 1067-72.

55. Freedman, G.M., Li, T., Anderson, P.R.*, et al.*, Health states of women after conservative surgery and radiation for breast cancer. Breast Cancer Res Treat, 2010. **121**(2): p. 519-26.

56. Johnson, F.R., Hauber, A.B., and Özdemir, S.J.V.i.H., Using conjoint analysis to estimate healthy‐year equivalents for acute conditions: an application to vasomotor symptoms. 2009. **12**(1): p. 146-152.

57. Locker, G.Y., Mansel, R., Cella, D.*, et al.*, Cost-effectiveness analysis of anastrozole versus tamoxifen as primary adjuvant therapy for postmenopausal women with early breast cancer: a US healthcare system perspective. The 5-year completed treatment analysis of the ATAC (‘Arimidex’, Tamoxifen Alone or in Combination) trial. 2007. **106**(2): p. 229-238.
